# Identifying diverse metal oxide nanomaterials with lethal effects on embryonic zebrafish using machine learning

**DOI:** 10.3762/bjnano.12.97

**Published:** 2021-11-29

**Authors:** Richard Liam Marchese Robinson, Haralambos Sarimveis, Philip Doganis, Xiaodong Jia, Marianna Kotzabasaki, Christiana Gousiadou, Stacey Lynn Harper, Terry Wilkins

**Affiliations:** 1 School of Chemical and Process Engineering, University of Leeds, Leeds, LS2 9JT, United Kingdom; 2 School of Chemical Engineering, National Technical University of Athens, 9 Heroon Polytechniou str. Zografou Campus, 15780 Athens, Greece; 3 School of Chemical, Biological, and Environmental Engineering, Oregon State University, Corvallis, Oregon, USA; 4 Department of Environmental and Molecular Toxicology, Oregon State University, Corvallis, Oregon, USA; 5 Oregon Nanoscience and Microtechnologies Institute, Eugene, Oregon, USA

**Keywords:** data augmentation, embryonic zebrafish, machine learning, nanosafety, nano-QSAR

## Abstract

Manufacturers of nanomaterial-enabled products need models of endpoints that are relevant to human safety to support the “safe by design” paradigm and avoid late-stage attrition. Increasingly, embryonic zebrafish (*Danio Rerio*) are recognised as a key human safety relevant in vivo test system. Hence, machine learning models were developed for identifying metal oxide nanomaterials causing lethality to embryonic zebrafish up to 24 hours post-fertilisation, or excess lethality in the period of 24–120 hours post-fertilisation, at concentrations of 250 ppm or less. Models were developed using data from the Nanomaterial Biological-Interactions Knowledgebase for a dataset of 44 diverse, coated and uncoated metal or, in one case, metalloid oxide nanomaterials. Different modelling approaches were evaluated using nested cross-validation on this dataset. Models were initially developed for both lethality endpoints using multiple descriptors representing the composition of the core, shell and surface functional groups, as well as particle characteristics. However, interestingly, the 24 hours post-fertilisation data were found to be harder to predict, which could reflect different exposure routes. Hence, subsequent analysis focused on the prediction of excess lethality at 120 hours-post fertilisation. The use of two data augmentation approaches, applied for the first time in nano-QSAR research, was explored, yet both failed to boost predictive performance. Interestingly, it was found that comparable results to those originally obtained using multiple descriptors could be obtained using a model based upon a single, simple descriptor: the Pauling electronegativity of the metal atom. Since it is widely recognised that a variety of intrinsic and extrinsic nanomaterial characteristics contribute to their toxicological effects, this is a surprising finding. This may partly reflect the need to investigate more sophisticated descriptors in future studies. Future studies are also required to examine how robust these modelling results are on truly external data, which were not used to select the single descriptor model. This will require further laboratory work to generate comparable data to those studied herein.

## Introduction

A variety of nanomaterial (NM)-enabled products have already been marketed [1–2] and there is considerable interest in the development of novel engineered nanomaterials (ENMs) for a variety of applications. Nanomedicine, including ENM-based therapeutic agents, nanocarriers (i.e., targeted drug delivery vehicles), diagnostic tools and medical devices, is a key application area [2–3]. However, as well as recognising the benefits associated with nanotechnology, it is also important to address potential negative impacts upon human health and the environment. Nanosafety concerns are reflected in international research efforts, such as the European Union’s NanoSafety Cluster [4] and associated research projects, such as BIORIMA [5], which has proposed a risk management framework for nanomaterials used in advanced therapeutic medicinal products and medical devices [6]. Indeed, in 2008, an iron oxide ENM-based magnetic resonance imaging (MRI) contrast agent (Feridex) was withdrawn from the market, following concerns regarding its observed side effects [2,7].

In spite of concerns around safety [7–9] and other challenges [10], there remains interest in developing novel metal oxide nanomaterials for various biomedical applications [10–12], as well as applications in other areas, such as in agriculture [13]. Nonetheless, the possibility that novel metal oxide ENMs developed for applications, such as biomedical applications, could be harmful to human health [8–9], means that there is a real need for developers of novel metal oxide ENMs and ENM-enabled products to introduce safety-by-design approaches. Due to the opportunity to reduce experimental costs and/or development timeframes and/or late-stage attrition, as well as the ethical, societal and regulatory [14–17] pressures towards reduced animal testing, there is considerable value in developing computational models that could reliably predict the toxic hazard of novel metal oxide ENMs, prior to experimental testing and, ideally, prior to synthesis, based upon synthesis-controlled, intrinsic characteristics of the ENMs. Indeed, the last decade has seen a significant number of published studies present quantitative structure–activity relationships (QSARs) for predicting the biological effects of ENMs, commonly known as nano-QSARs, based upon calculated and/or measured variables (descriptors) related to their intrinsic or extrinsic (i.e., depending on the exposure medium) physicochemical characteristics [18–20].

However, concerns have been raised regarding the human health relevance of the endpoints modelled in many nano-QSAR studies [21]. There is a need for models that can predict human safety relevant endpoints. Increasingly, there is interest in using (embryonic) zebrafish (*Danio Rerio*) as experimental test subjects to assess potential human safety concerns of chemicals [22] and materials, including nanomaterials [23–24]. It is argued that embryonic zebrafish provide “the power of whole-animal investigations […] with the convenience of cell culture” [25] and that “zebrafish exhibit remarkable similarity to other high-order vertebrates including humans” [23]. Hence, the Nanomaterial Biological Interactions (NBI) Knowledgebase [26], a publicly available database of ENM embryonic zebrafish test results for various lethal and sub-lethal biological endpoints, determined at a range of test concentrations, is a valuable resource for developing human safety relevant nano-QSAR models. Importantly, all biological data from this database were obtained in the same laboratory (Harper Laboratory, Oregon State University), with minimal, clearly documented, variations in experimental conditions, and were linked to comparable physicochemical characterisation data.

However, the first published modelling studies of NBI Knowledgebase data treated the characterisation data, used as input to predictive models, in a simple fashion [23,27]. Specifically, in addition to using other qualitative and quantitative physicochemical characteristics (such as average primary particle size), these studies only used the names of chemical components, that is, the core, surface functional groups (FGs) or the shell, as descriptors. This is restrictive, as generalization for nanomaterials with similar but non-identical chemical compositions is not possible. Subsequent studies only developed local models for ENMs with nominally the same core material, for instance, gold or zinc oxide, where only the variation in the chemical composition of surface features was encoded using generalisable descriptors [28–29]. Most recently, Gousiadou et al. [30] developed the first models for diverse nanomaterial data from the NBI Knowledgebase where all chemical composition was encoded using calculated, numerical descriptors allowing for greater generalisation. Nonetheless, all of these previous studies were concerned with regression models for predicting the numerical biological response at a single test concentration. In addition to these modelling studies, Karcher et al. [31] reported analyses of trends in NBI Knowledgebase biological effects data with various ENM characteristics.

In the current work, classification models were developed to classify coated or uncoated metal oxide nanomaterials as lethal or non-lethal, based upon whether statistically significant lowest observed effect levels (LOELs) [32] for lethality, or excess lethality, in embryonic zebrafish were detectable at test concentrations up to 250 parts per million (ppm). Models were developed using data measured at 24 and 120 hours post-fertilisation (hpf) for a diverse set of metal oxide ENMs. Effects at 120 hpf were assessed with respect the embryonic zebrafish surviving after 24 hpf, that is, LOEL values for excess lethality at 120 hpf were derived, to reduce the potential mixing of mortality arising via different exposure routes [31]. The data and characterisation variables used as the basis for descriptors were based upon the previously reported analyses of the NBI Knowledgebase by Karcher and co-workers [31].

In contrast to most previously published modelling studies of the NBI Knowledgebase (discussed above), we developed toxicity models using a variety of particle characteristics and variables calculated based upon the chemical composition of the core and organic surface components, which allow for greater generalisability than simply using the names of the chemical constituents directly as variables, for a diverse set of coated and uncoated inorganic nanomaterials. Moreover, in contrast to all previously published modelling studies of NBI Knowledgebase data, we developed classification models based upon LOEL values, taking into account statistical significance of observed effects vs control values, rather than simply modelling the data at different test concentrations. (This means we are modelling a less noisy, more reliable endpoint.) A further novel aspect of our study is that we explored two data augmentation approaches which, to the best of our knowledge, have never previously been applied in published nano-QSAR research, as a means of addressing the widely known issues with limited availability of suitable data for nano-QSAR development [20,33–34]. These approaches were a novel framework that is analogous to that of Kim et al. [35], as well as an approach closely linked to Cortes-Ciriano and co-workers [36]. Finally, we explored whether comparable modelling results could be obtained using a simple single descriptor model, in contrast to the multi-descriptor models explored in previous studies of NBI Knowledgebase datasets.

## Results and Discussion

### Overview of data and modelling studies

A dataset comprising 44 ENMs was derived from the NBI Knowledgebase to support the development of models for classifying coated and uncoated metal oxide ENMs as toxic or non-toxic, according to two distinct categorisations based upon mortality data determined at 24 or 120 hpf for embryonic zebrafish continuously exposed to the ENMs via fish water test medium [31]. (One dataset entry corresponded to an ENM with a silicon dioxide core. Whilst silicon dioxide is, strictly speaking, a metalloid oxide, it is considered a metal oxide according to the NBI Knowledgebase terminology and seminal nano-QSAR work [18].) Both endpoints were treated as binary classification variables, with ENMs considered toxic if a LOEL value of at most 250 ppm (mass-based concentrations) could be derived or, otherwise, non-toxic. Any materials not tested at a maximum concentration of 250 ppm were removed to ensure consistency.

**Figure 1:**
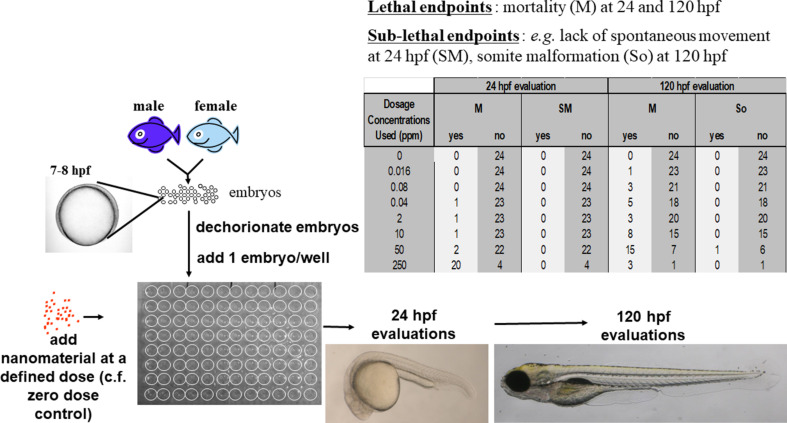
The laboratory method used to obtain the raw counts data for the lethal and sub-lethal endpoints evaluated for each tested engineered nanomaterial (ENM) for which data are reported in the Nanomaterial Biological-Interactions (NBI) Knowledgebase. At the start of the experiment, at around 7–8 hpf, a fixed number of embryos are exposed to a fixed dose of ENM. At 24 hpf, for each tested dose, including the zero-dose control, the endpoint is observed (“yes”) or not observed (“no”) for a certain number of those embryos. At 120 hpf, observations are made for the embryos that survived up to 24 hpf, that is, these raw counts data correspond to excess effects occurring in the period of 24–120 hpf.

**Figure 2:**
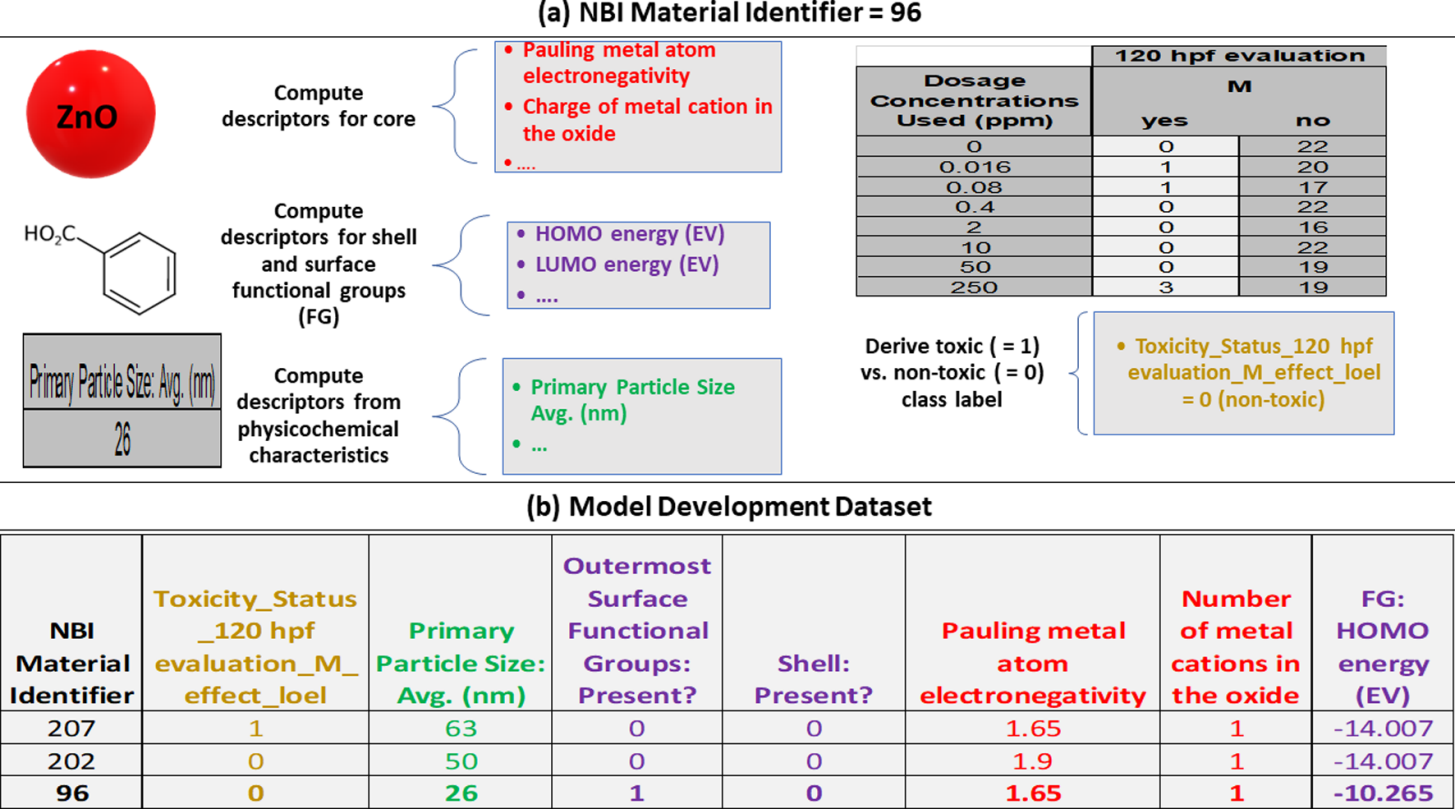
The transformation of the raw biological counts data, illustrated here for excess lethality at 120 hpf, and ENM core and surface chemical composition data, along with other reported physicochemical characteristics, into the modelled categorical endpoint data and the descriptors used to model them respectively. (a) This process is illustrated for the ENM corresponding to NBI Material Identifier 96 (coated zinc oxide with benzoic acid outermost surface functional groups). (b) A subset of the resultant model development dataset is shown, including three of the modelled ENMs and a subset of the descriptors used to model excess lethality at 120 hpf. Dummy values were assigned for the shell and/or outermost surface functional group descriptors if no shell and/or outermost surface functional groups were present.

Figure 1 summarizes the laboratory protocol used to derive the raw data reported in the NBI Knowledgebase regarding the occurrence frequency of lethality and sub-lethal effects at each of the tested ENM concentrations and the zero-dose control group [25,28,31]. Figure 2 summarizes the procedure used in the current study to convert the raw observations at either 24 or 120 hpf into binary classification labels, meaning toxic (= 1) vs non-toxic (= 0) for observations of lethality, for each combination of endpoint and ENM.

Modelled effects at 120 hpf were assessed with respect the embryonic zebrafish surviving after 24 hpf, to reduce the potential mixing of mortality arising via different exposure routes, as the oral exposure route becomes possible between 24 and 120 hpf [31]. This means that the modelled lethality data at 120 hpf refers to fish embryos that were alive at 24 hpf but that were dead at 120 hpf. (More generally, all effects, both lethal and sub-lethal, at 120 hpf were evaluated relative to the zero-dose control group based upon the embryos that survived at 24 hpf in both the control and dosed groups.) Hence, the modelled lethality data reported at 120 hpf, derived from the raw counts data, may be considered “excess lethality”.

Two different machine learning algorithms were applied to try and learn relationships between either the 24 hpf lethality or 120 hpf excess lethality data, converted to a binary response, and input variables characterizing the chemical composition and particle characteristics of the ENMs. As noted above, these binary responses were toxic (a LOEL for the modelled lethal effect was detected) vs non-toxic (a LOEL for the modelled lethal effect was not detected). The input variables are termed “descriptors” in keeping with standard nano-QSAR terminology [19]. The relationships between the biological response data and the descriptors are learnt by applying the models to the available experimental data, or a subset of it, linking the binary response variable to be modelled and the descriptors, known as a “training set” [19].

The algorithms used were the SciKit-Learn [37] adaptations of Random Forest [38–40] and logistic regression [41–42]. The models built using both algorithms generate a score between zero and one for an ENM, which represents the confidence of the model that it belongs to the toxic category, with a score greater than 0.5 resulting in a “toxic” prediction and lower scores resulting in a “non-toxic” prediction. Whereas logistic regression builds a parametric model, which assumes each descriptor contributes independently to the prediction, Random Forest builds a non-parametric model, comprising a set of decision trees constructed via randomly sampling the available training data, which makes no such assumptions.

The different machine learning modelling approaches (meaning combinations of learning algorithm, descriptors and data augmentation approaches), were evaluated via a fivefold cross-validation (5-CV) framework on folds that were consistent across both endpoints and modelling approaches. By fivefold cross-validation, we mean the available model development data were randomly partitioned into five sets, or “folds”, of roughly equal numbers of ENMs, and a model built using four out of the five folds was evaluated on the remaining fold, with performance statistics averaged across all folds.

All of the individual cross-validation results presented herein were generated using a nested cross-validation framework, sometimes even referred to as “external cross-validation”, such that any selection of model parameters was carried out independently for each cross-validation split, using the cross-validation training set alone, to minimise the risk of optimistic bias in the model performance statistics [43–44]. (The one exception to this was the use of the entire model development set to select dummy values for surface component descriptors, but this is not expected to have any influence on the results with Random Forest [38], and the weak importance assigned to these descriptors indicates this was not a cause of significant optimistic bias for any method.) However, it should be acknowledged that the exploration of different modelling approaches, especially the final selection of a single descriptor model based upon descriptor importance analysis performed using all 44 ENMs, might have resulted in optimistic bias.

The descriptors (Figure 2) used to model these endpoints corresponded to a variety of particle characteristics and variables calculated based upon the chemical composition of the core and, where applicable, organic surface components, including shell and outermost surface functional groups [31]. (All calculations treated the core material and surface components independently, that is, the descriptors were calculated for the surface components treated as free molecules.) Since shell and/or surface functional groups were not present for all ENMs (i.e., the ENM could be uncoated), it was necessary to assign dummy values for the molecular descriptors where the corresponding components did not exist (i.e., where the descriptors were not applicable). However, the dataset was selected such that, in contrast to non-applicable dummy values [45], there were no missing values for numeric variables. Unless mentioned otherwise, all numeric descriptors were mean centred and scaled to unit variance, based upon the training set distributions. Non-numeric particle characteristics (e.g., core shape) were one-hot-key encoded, that is, each value encountered in the training set (e.g., “cylindrical”, “unknown”) was treated as a unique descriptor, with values of one and zero assigned if the ENM had and did not have the specified value for that characteristic respectively.

**Figure 3:**
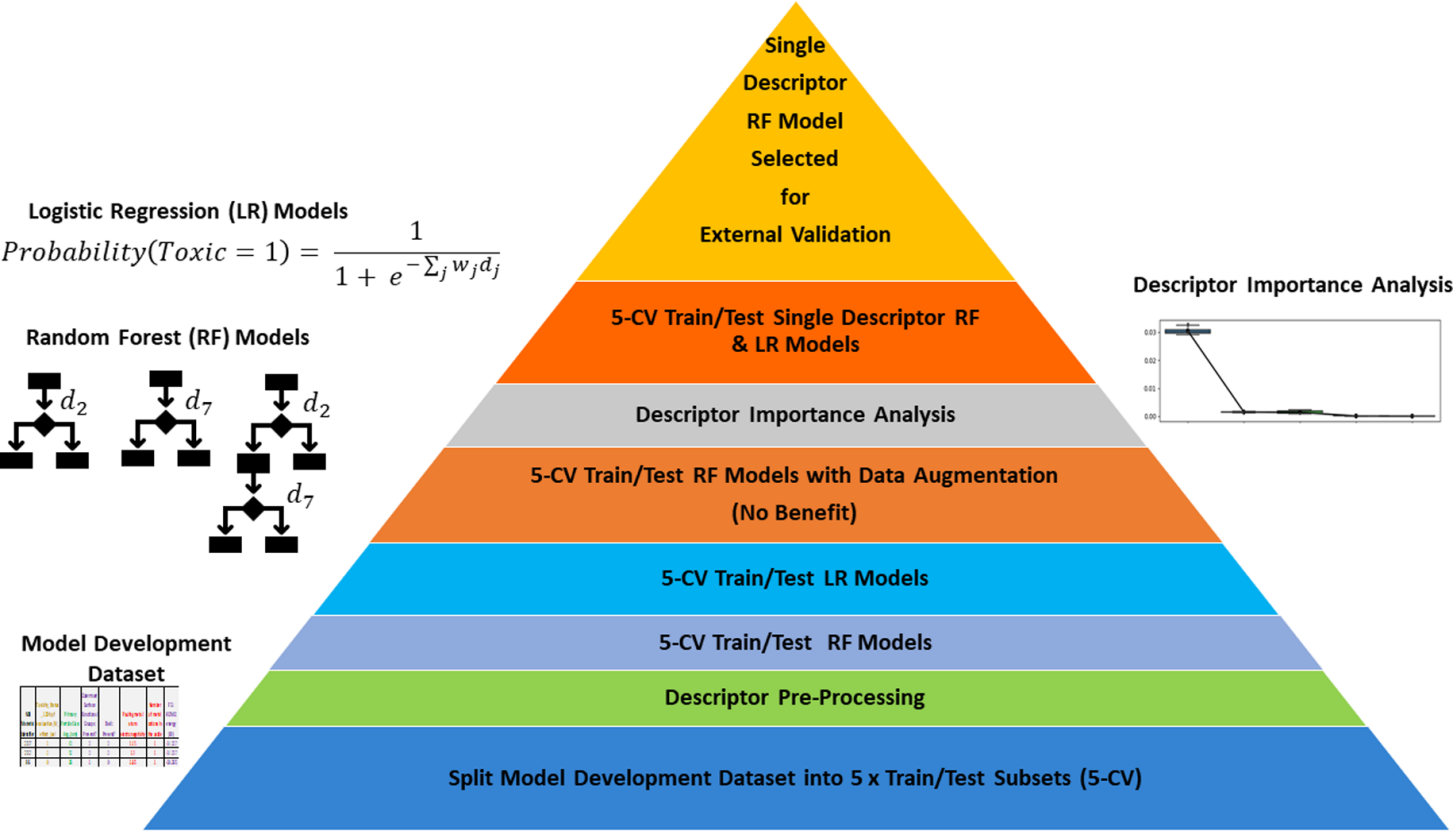
A summary of all modelling work carried out using the model development dataset reported herein. A schematic of the Random Forest modelling technique is provided, illustrating how different descriptors (*d_j_*, *j* = 1, 2, 3, …) are selected at each node to best separate toxic from non-toxic ENMs based upon random subsets of the available training data and, at each node, the available descriptors. In reality, the depth of the trees may be greater than shown here and several hundred trees were investigated, with the precise number being one of the investigated hyperparameters tuned using the training data. For logistic regression, each descriptor is assumed to make an independent contribution, according to corresponding weights (*w_j_*), to the probability of the ENM being toxic. Both kinds of model, using either all available descriptors or a single descriptor identified via subsequent descriptor importance analysis based upon the entire model development dataset, were evaluated on the model development set using nested fivefold cross-validation (5-CV) to minimize optimistic bias in the evaluation of model performance – although the estimated performance of the selected single descriptor model may suffer more from optimistic bias. Unless noted otherwise, data augmentation was not used to enhance the data used for training the models.

A summary of all modelling carried out in the current work is presented in Figure 3. In addition to the exploration of different machine learning algorithms, data augmentation approaches and the construction of simple models based upon the most significant descriptor were investigated.

**Figure 4:**
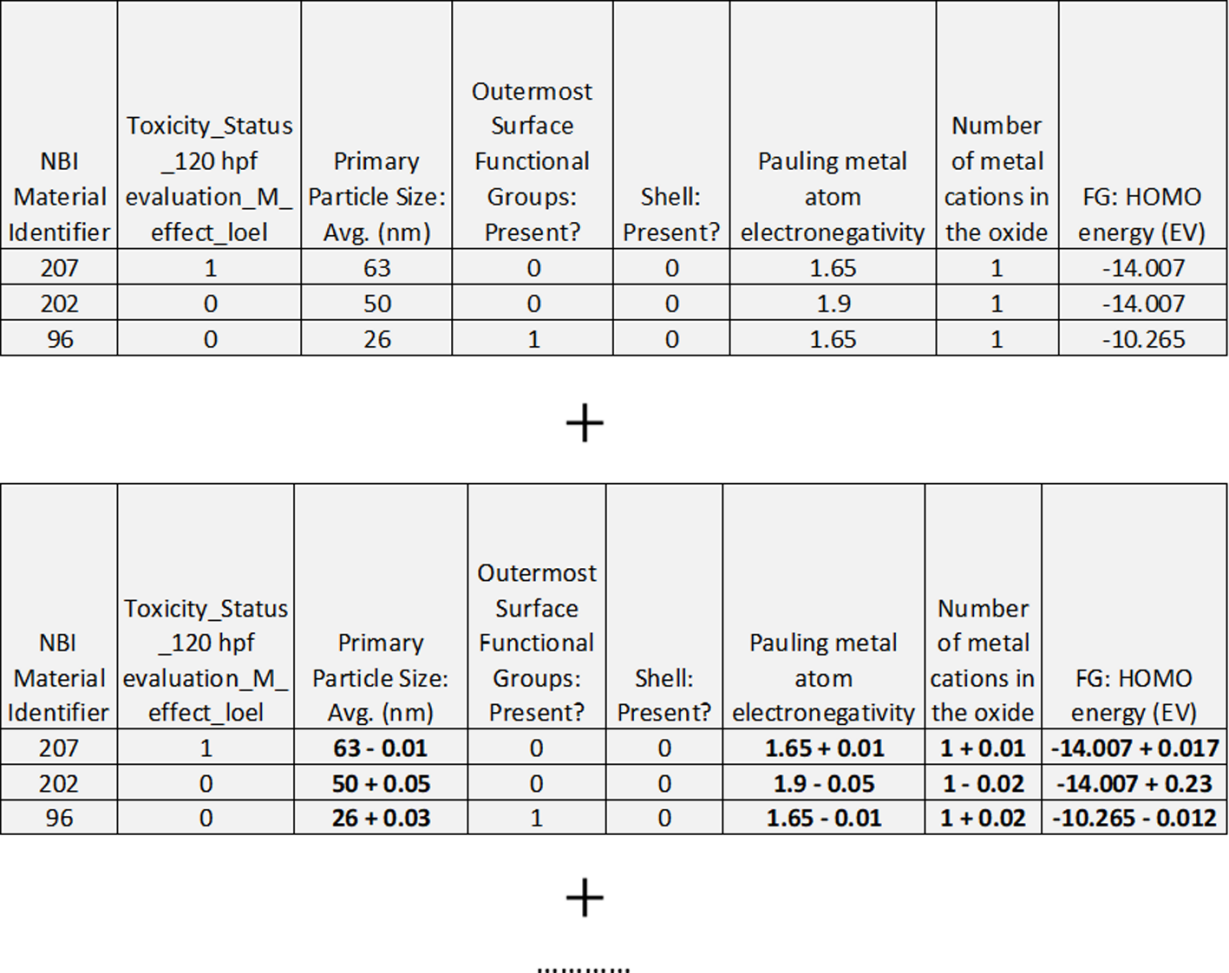
Data augmentation using the “noised training set replication” paradigm. Top: a subset of the available training set ENM samples and the corresponding descriptors is shown. Bottom: the addition of one or more “noised” replicates of the training set, including the perturbation of numeric descriptors using Gaussian noise in bold, is shown.

**Figure 5:**
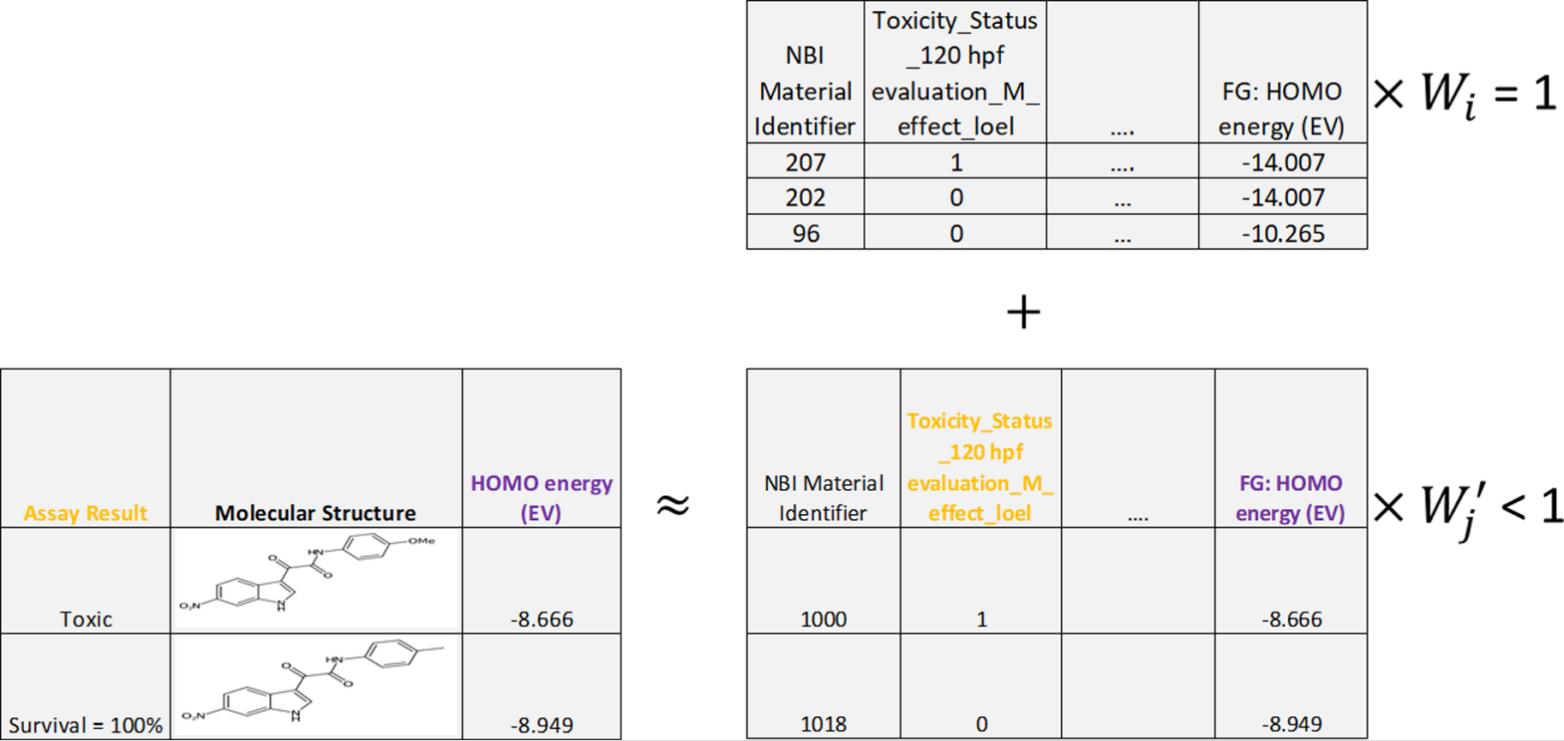
Data augmentation using the “weighted alternative samples” paradigm, where the alternative samples were derived from molecular toxicity data reported in the ChEMBL database corresponding to whether mortality was (“toxic”) or was not (“survival = 100%”) observed after three days, when compounds were tested at 5 μM at 48 hpf on zebrafish. Top: a subset of the available training set ENM samples and the corresponding descriptors is shown. The weighting of these true samples during training (*W_i_*) was set to 1.0. Bottom: the addition of some of the available pseudo-ENM samples is shown, where these pseudo-ENM samples were derived by treating the compounds as outermost surface functional groups from ENM samples, with all other ENM descriptors set to dummy values assigned to inapplicable descriptors. The weighting of these pseudo-samples during training (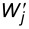) was set to a value less than 1.0.

**Figure 6:**
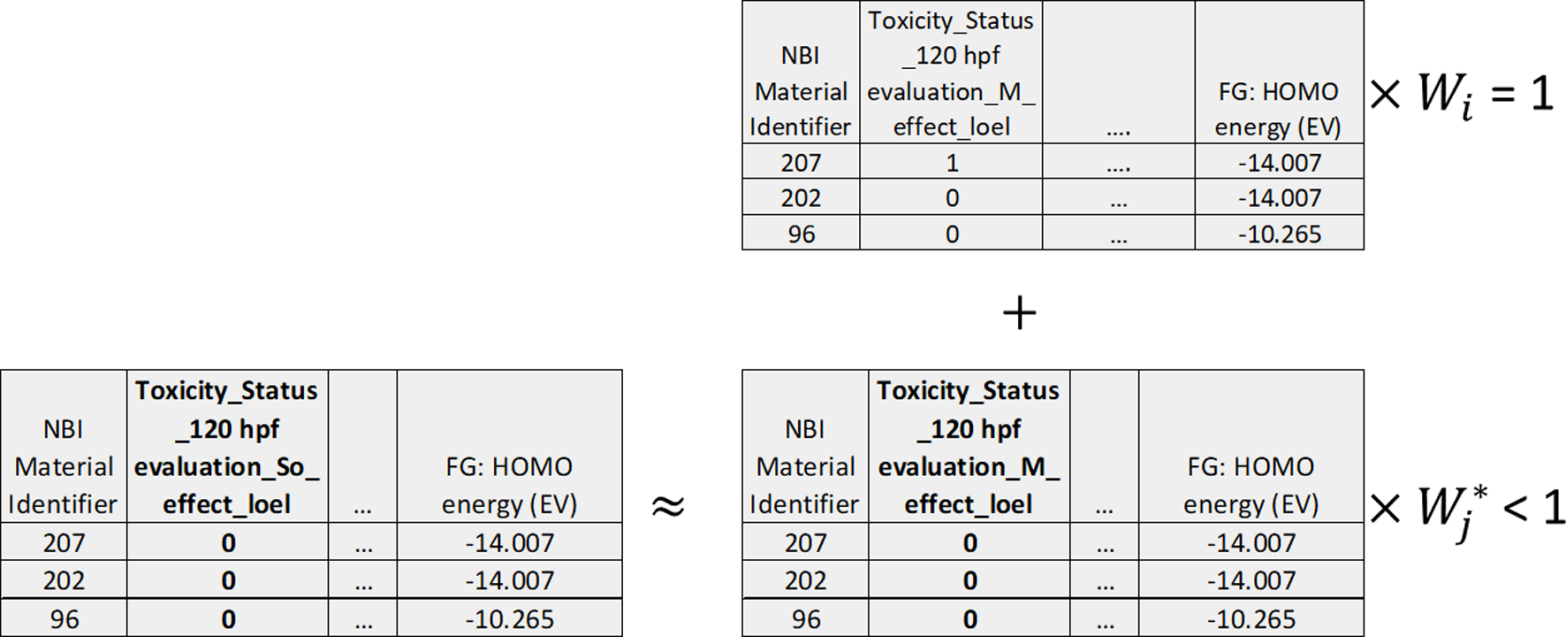
Data augmentation using the “weighted alternative samples” paradigm, where the alternative samples were the original ENM samples from the training set with the binary response values toxic (= 1) vs non-toxic (= 0), corresponding to lethality at 24 hpf or excess lethality at 120 hpf, replaced with the binary response values active (= 1) vs inactive (= 0), corresponding to one of the measured sub-lethal endpoints. Top: a subset of the available training set ENM samples and the corresponding descriptors is shown. The weighting of these true samples during training (*W_i_*) was set to 1.0. Bottom: the addition of some of the alternative ENM samples is shown. The weighting of these samples during training (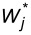) was set to the estimated correlation between the modelled lethal endpoint and the sub-lethal endpoint for which the data were treated as additional data for the lethal endpoint.

Two data augmentation paradigms were investigated here (see below in Figures 4–6). To the best of our knowledge, neither paradigm has previously been investigated in nano-QSAR. Both relied upon supplementing the training sets with pseudo-additional data for the modelled lethality endpoints.

The first paradigm (“noised training set replication”, Figure 4) was to add multiple copies of the original training set, with descriptor values for each new copy being randomly perturbed. This was successfully applied by Cortes-Ciriano and Bender to improve predictive performance for regression QSAR modelling of molecular bioactivity [36]. The effectiveness of this approach relies upon the similarity principle, that is, the assumption that a small perturbation in structure, corresponding to a small perturbation in descriptors, will lead to a small change in biological activity [36]. In the current context, it is assumed that the toxicity classes would be unchanged for these small perturbations. Descriptors corresponding to qualitative particle characteristics, such as particle shape, or the presence or absence of a shell or surface functional groups, were not randomly perturbed, as these cannot vary by small amounts.

The second paradigm (“weighted alternative samples”) was to add data for some other endpoint to the training set and assign the weight of the new samples, that is, their importance during training, based upon the (estimated) similarity of the new endpoint to the original endpoint actually being modelled. This novel paradigm may be considered analogous to the data augmentation approach previously applied by Kim et al. [35] to model synthesis routes for inorganic materials.

Here, the original endpoint actually being modelled was always one of the previously described binary response variables (toxic vs non-toxic) based upon whether a LOEL was identified for lethality at 24 hpf or, alternatively, excess lethality at 120 hpf. When the new samples were added to the training set, their binary response variable corresponding to the new endpoint (either toxic vs non-toxic or active vs inactive, depending upon whether the new endpoint corresponded to a lethal or sub-lethal response) was treated as if it was a new set of values for the original (excess) lethality endpoint being modelled. This was hypothesized to be justified since the new and original endpoint were expected to be related. However, since the new endpoint values were not actually values for the original endpoint being modelled, the weight given to the new samples was always set to a value less than 1.0, whilst the weight assigned to the original samples was always set to 1.0. These sample weights determined the extent to which the different samples were able to influence the training of the Random Forest models, that is, assigning sample weights of zero would have been equivalent to not including the new samples [46].

Two variations on this second paradigm were investigated. (1) The data augmentation samples were data points for zebrafish mortality in response to treatment with molecular compounds at 5 μM at 48 hpf, measured after three days (Figure 5). These data were obtained from ChEMBL [47–48] (assay ID = CHEMBL1913666 [49], retrieved on 20th November 2019). They were treated as pseudo-ENM data samples, with the modelled toxic and non-toxic classes being assigned on the basis of whether the data were reported as “toxic” vs “survival = 100%”, respectively. Molecular descriptors were computed as per the treatment of ENM surface components and dummy values were assigned for all other descriptors. The similarity weighting of these samples was treated as a tunable hyperparameter between 0.1 and 0.9. (2) The new samples were actually the original samples save for the fact that the modelled mortality endpoint (toxic vs non-toxic classes) for the replicated samples was substituted by one of the corresponding sub-lethal endpoint values (active vs inactive classes), as shown in Figure 6. If the sub-lethal endpoint corresponded to an effect measured at 120 hpf and the lethality endpoint being modelled corresponded to 24 hpf, these samples were not considered for data augmentation. Here, the weights of the data augmentation samples were set to the Matthews correlation coefficient (MCC) between the modelled mortality endpoint and the substituted endpoint for the data augmentation samples, computed separately across each training set, or zero if this was negative. Whilst it could reasonably be argued that these other endpoint data would not be available for untested, or hypothetical, ENM samples for which future predictions are desired, this may be considered a proof-of-concept of this novel data augmentation paradigm, removing any complications associated with using ENMs with dissimilar physicochemical/particle characteristics as the data augmentation samples.

Here, it is important to emphasize that the sub-lethal endpoints, or other proxy endpoints considered in the “weighted alternative samples” paradigm, were not specifically modelled per se. Rather, they were treated as if they were additional values for the lethal endpoint being modelled during training and given lower weight during training to reflect the fact that the sub-lethal and lethal endpoints do not perfectly correlate. In all cases, the performance of the models was assessed purely on their ability to predict the values of the original lethal endpoint being modelled in the original cross-validation test sets.

### Full details of data and modelling studies

Full details regarding dataset selection, estimation of LOELs, descriptors, dummy values, descriptor scaling, modelling (including selection of model hyperparameters and descriptors as applicable) and analysis methods are provided under the Experimental section of this article. A step-by-step explanation of how to reproduce our results, using the open source code we have made available, is provided in Supporting Information File 1.

#### Random Forest cross-validated classification results without data augmentation

The overall performance of the Random Forest models for both endpoints, as assessed on a consistent set of cross-validation folds, is reported in Table 1 in terms of the balanced accuracy, Matthews correlation coefficient (MCC) and area under the receiver operator characteristic curve (AUC). (Recall and precision statistics, indicating the performance for individual categories, are reported in an expanded version of this table in Supporting Information File 1, Table S1.) Clearly, the average predictive performance for lethality (mortality) at 24 hpf appears notably worse than predictions of excess lethality at 120 hpf. (Indeed, the average performance for modelling of the 24 hpf endpoint appears comparable to what would be expected from random guessing, with values of 0.50, zero and 0.50 expected for the balanced accuracy, MCC and AUC, respectively, in this scenario [50–52].) Similar, albeit marginally better, findings were obtained when the model for the 24 hpf endpoint was cross-validated using folds identified using stratification based upon the 24 hpf endpoint (see Supporting Information File 1, Table S2), rather than the same folds identified using stratification based upon the 120 hpf endpoint for consistent comparison of results, as per Table 1.

**Table 1:** Average performance statistics obtained from cross-validation of Random Forest (RF) classification models and logistic regression (LR) models on consistent folds of the model development dataset. All models were built without data augmentation, using all calculated descriptors, unless noted otherwise. All results were obtained via tuning hyperparameters separately for each cross-validation training set, that is, the multiple descriptor results are not expected to suffer from significant optimistic bias. However, the single descriptor (Pauling metal atom electronegativity) models were developed following descriptor importance analysis using the entire model development dataset. The performance in terms of each figure of merit is summarised as the arithmetic mean ± standard error [median]. (The standard error of the mean is an underestimate of the uncertainty, as the cross-validation results are not entirely independent.) BA = balanced accuracy, MCC = Matthews correlation coefficient. AUC = area under the receiver operator characteristic (ROC) curve.

Lethality endpoint	Model	BA	MCC	AUC

24 hpf	RF	0.44 ± 0.05 [0.50]	−0.12 ± 0.10 [0.00]	0.36 ± 0.10 [0.43]
120 hpf (excess lethality)	RF	0.71 ± 0.08 [0.67]	0.45 ± 0.17 [0.33]	0.76 ± 0.09 [0.71]
	LR	0.74 ± 0.11 [0.60]	0.48 ± 0.21 [0.22]	0.70 ± 0.14 [0.67]
	RF (single descriptor)	0.74 ± 0.09 [0.75]	0.52 ± 0.20 [0.65]	0.79 ± 0.08 [0.83]
	LR (single descriptor)	0.73 ± 0.10 [0.67]	0.43 ± 0.19 [0.33]	0.73 ± 0.11 [0.63]

The greater difficulty associated with modelling the 24 hpf endpoint might possibly reflect different systemic exposure routes and the failure of the descriptors to properly account for factors affecting dermal penetration. Whilst the experimental setup involved continuous waterborne exposure of the embryonic zebrafish to the ENMs dispersed in fish water medium and dermal penetration is typically observed as the main route of systemic exposure up to 120 hpf, the appearance of swallowing around 72 hpf allows for toxicity via an oral exposure route to manifest prior to 120 hpf but not at 24 hpf [31]. However, it is also possible that the observed difference in modelability at 24 and 120 hpf could reflect differential susceptibility of the embryos to specific toxic modes of action [31].

Based upon the poor modelability of the 24 hpf endpoint observed here, subsequent discussion focuses upon modelling of the 120 hpf endpoint.

#### Data augmentation techniques fail to clearly improve performance

**Figure 7:**
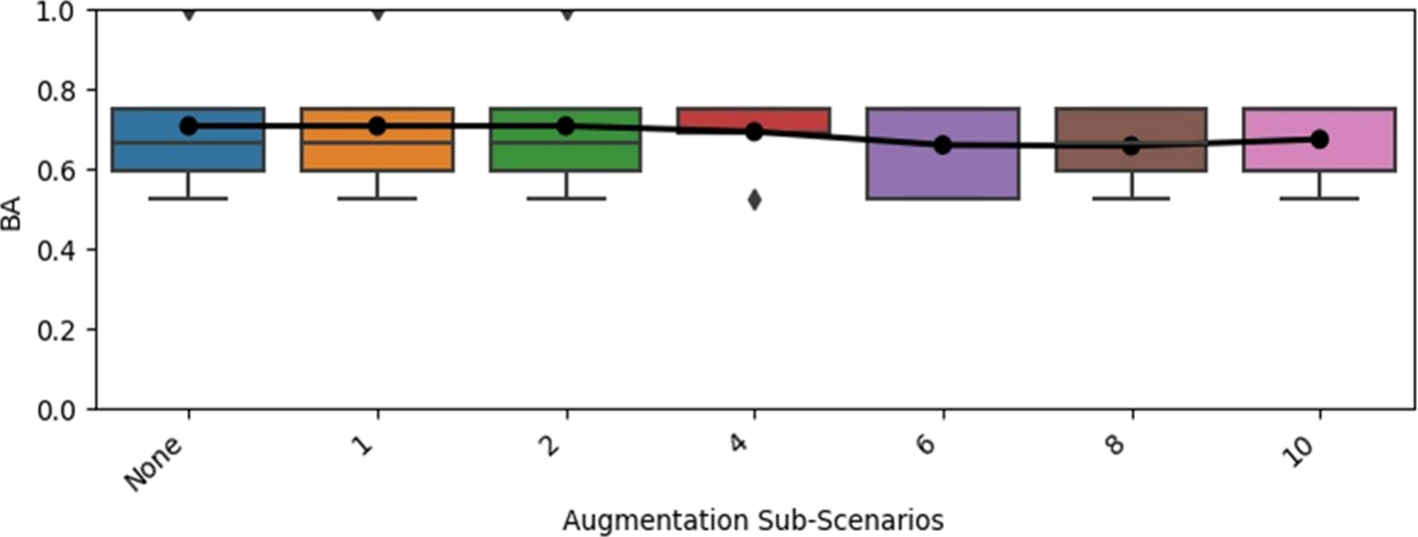
Nested cross-validated balanced accuracy (BA) obtained with multiple descriptor Random Forest models for the 120 hpf excess lethality endpoint when the cross-validation training sets were supplemented with multiple noised replications (1–10 replications, with certain numbers skipped in keeping with literature precedence [36]) of themselves, compared to the results obtained with no data augmentation (“None”). (The results with no data augmentation were reported in Table 1). The results across all test folds are summarized in terms of a boxplot, with the arithmetic mean result superimposed. The whiskers extend to data points lying up to 1.5 times the interquartile range above and below the upper and lower quartiles, respectively. The triangles/diamonds represent outlier results, which are higher and lower than the upper and lower whiskers, respectively.

**Figure 8:**
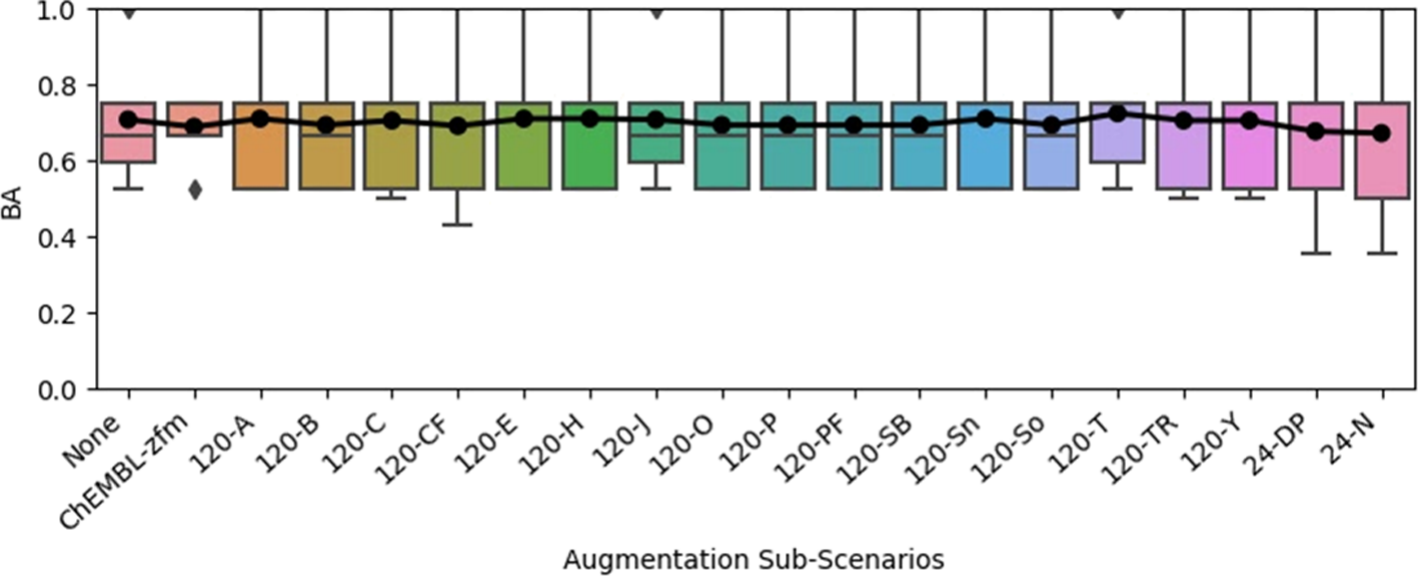
Nested cross-validated balanced accuracy (BA) obtained with multiple descriptor Random Forest models for the 120 hpf excess lethality endpoint when the cross-validation training sets were supplemented with weighted alternative samples, compared to the results obtained with no data augmentation (“None”). (The results with no data augmentation were reported in Table 1). Other than where molecular zebrafish lethality data from ChEMBL were treated as pseudo-coated ENM samples, all other alternative samples corresponded to the cross-validation training set with the modelled endpoint substituted with one of the sub-lethal endpoints. (See section “Endpoint abbreviations” under the Experimental section for an explanation of the 24 and 120 hpf sub-lethal endpoint abbreviations.) The results across all test folds are summarized in terms of a boxplot, with the arithmetic mean result superimposed. The whiskers extend to data points lying up to 1.5 times the interquartile range above and below the upper and lower quartiles, respectively. The triangles/diamonds represent outlier results, which are higher and lower than the upper and lower whiskers, respectively.

Across all augmentation scenarios, for both the 24 hpf lethality and 120 hpf excess lethality endpoints, no statistically significant increases at the 5% level were observed in the mean cross-validated overall performance, whether measured in terms of balanced accuracy, MCC or AUC. Indeed, this was found to be the case even prior to adjusting the *p*-values to account for multiple testing considerations. For illustrative purposes, the trends observed for the “noised training set replication” (Figure 7) and “weighted alternative samples” (Figure 8) data augmentation paradigms are shown for modelling of the 120 hpf excess lethality endpoint with predictive performance characterised in terms of the balanced accuracy. The corresponding plots for the 24 hpf lethality endpoint and other overall performance measures (MCC and AUC) are shown in Supporting Information File 1, Figures S1–S10.

The failure of the “noised training set replication” paradigm to improve model performance (Figure 7) is surprising, given that this has been successfully applied in various other scenarios [36]. It is possible that the presence of activity cliffs may have contributed to this finding [36]. Another possibility is that the perturbations were typically too small to make any difference to the outcome of a Random Forest prediction, for which changes in descriptor values must shift an instance from one side of a tree split point to another in order to result in any change in the outcome. However, the analysis of Cortes-Ciriano and Bender [36], whilst not identical to the analysis performed here, found that adding noise, of the same magnitude considered in the current study, to the descriptors did allow data augmentation to improve the performance of Random Forest models.

**Figure 9:**
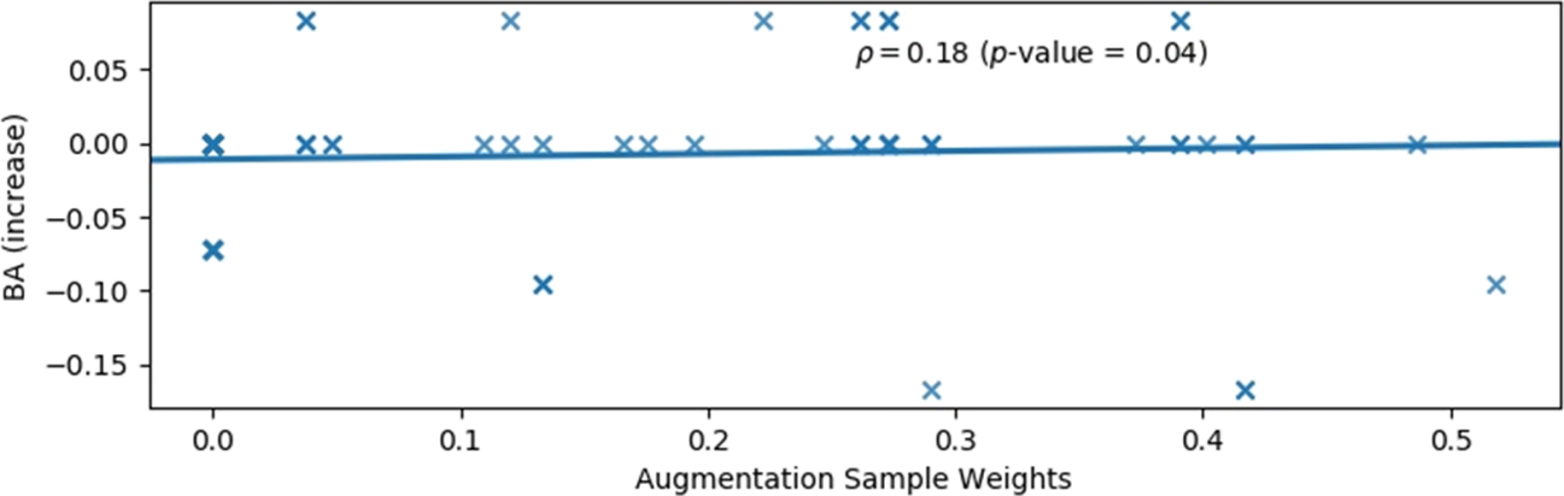
The change in cross-validated balanced accuracy (BA), with results shown for individual folds, obtained with Random Forest models for the 120 hpf excess lethality endpoint when the cross-validation training sets were supplemented with alternative samples, derived via replacing the training set sample endpoint values with the endpoint values for sub-lethal endpoints measured for the same materials, plotted against the weight given to these alternative samples. Here, the weight assigned was either the MCC correlation measure, if this was positive, or zero. The Spearman rank correlation coefficient is shown, along with the corresponding one-tail *p*-value for the null hypothesis that this is zero, that is, that there is no correlation between the change in performance and the relationship between the modelled and sub-lethal endpoint data used for data augmentation.

The failure of the “weighted alternative samples” approach to improve model performance (Figure 8) possibly reflects insufficient similarity between the data used for augmentation and the modelled data. It was typically observed that, across the cross-validation training sets, the MCC between the modelled lethal endpoint and the sub-lethal endpoint, used as a proxy when the training samples were augmented with themselves, was less than 0.5. However, negligible rank correlation was observed between the data augmentation weighting (i.e., the MCC between the modelled and sub-lethal endpoint, for positive values, or zero otherwise) and the change in model performance. This is illustrated by Figure 9. The corresponding plots for the 24 hpf lethality endpoint and other performance measures are shown in Supporting Information File 1, Figures S11–S15. Indeed, it should also be noted that the extent to which a correlation would be expected is unclear, since augmenting the training set with an exact copy of the original training set would presumably correspond to adding redundant information, yet would correspond to an MCC of one.

Since data augmentation did not clearly improve performance with Random Forest, no further consideration was given to the use of this modelling strategy.

#### Logistic regression cross-validated classification results

Logistic regression [41–42] modelling results were also obtained for the 120 hpf excess lethality endpoint using all descriptors and the same cross-validation folds as per the nested cross-validation results reported for Random Forest. As can be seen from Table 1, the overall performance with logistic regression is comparable to Random Forest for modelling of excess lethality at 120 hpf.

#### Toxicologically significant nanomaterial characteristics

**Figure 10:**
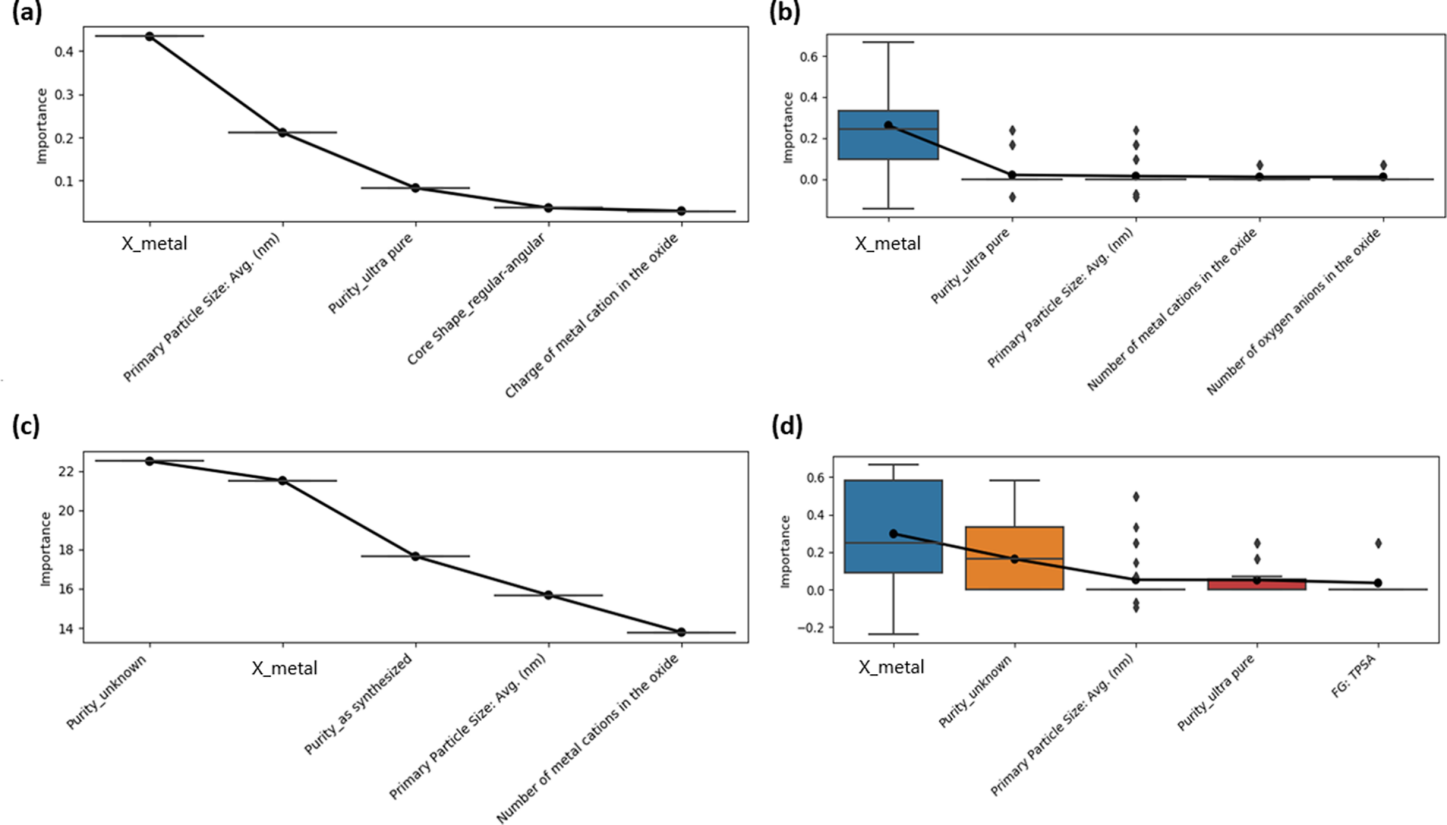
Variable importance estimates for the five most important variables (highest mean importance values) in the original multiple descriptor models, without data augmentation, of excess lethality at 120 hpf. “X_metal” denotes the Pauling metal atom electronegativity descriptor. See section “Descriptor calculations” under the Experimental section for detailed explanations of the names of the other descriptors. (a) Original Gini importance values for a single Random Forest model trained on the entire model development dataset, with hyperparameters tuned via cross-validation. (b) Distributions of permutation variable importance values (change in balanced accuracy after permutation) across ten random permutations of the descriptor values in the validation fold and five folds, for Random Forest models cross-validated on the model development set, with descriptor scaling and hyperparameter tuning repeated for each cross-validation training set. (c) Original coefficient magnitude importance values for a single logistic regression model trained on the entire model development dataset, with partial hyperparameter tuning via cross-validation. (d) Distributions of permutation variable importance values (change in balanced accuracy after permutation) across ten random permutations of the descriptor values in the validation fold and five folds, for logistic regression models cross-validated on the model development set, with descriptor scaling and hyperparameter tuning repeated for each cross-validation training set. The mean variable importance measures (black circles) are superimposed upon the boxplots of the distributions.

Analyses of the descriptors that were most related to the 120 hpf excess lethality endpoint are presented in Figure 10. The Pauling electronegativity of the metal atom corresponding to the metal cation in the ENM core was amongst the top two descriptors for both Random Forest and logistic regression. Indeed, for a permutation importance measure, reflecting the reduction in cross-validated balanced accuracy upon random permutation of the descriptor values in the validation folds [53–54], the Pauling metal atom electronegativity [55] was the most important descriptor for both modelling methods. Albeit this finding was more robust with Random Forest.

**Figure 11:**
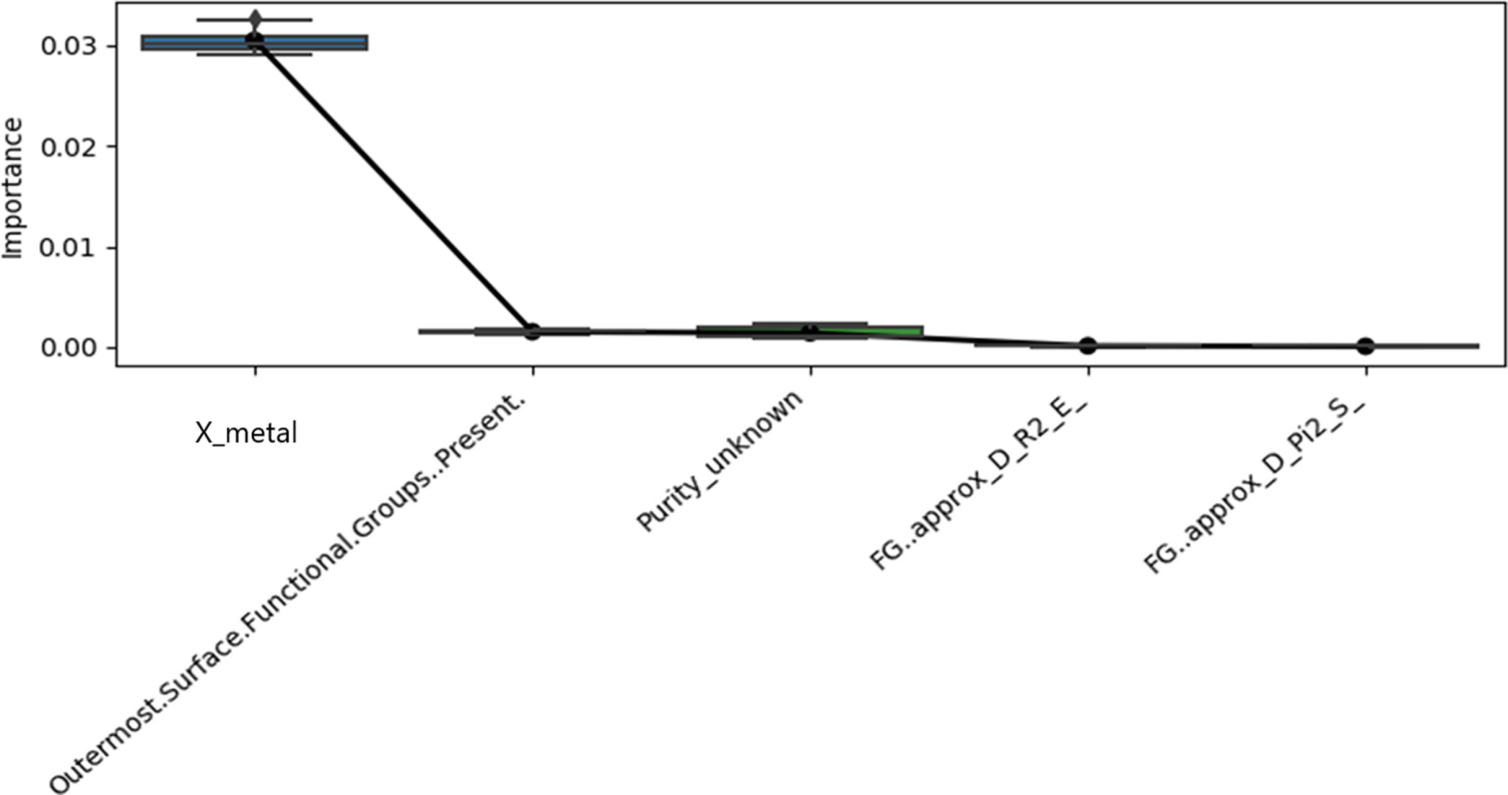
Distributions of conditional variable importance estimates for the five most important variables (highest mean importances) across ten Cforest multiple descriptor models, built without data augmentation on the original model development dataset, of excess lethality at 120 hpf. The mean variable importance measures (black circles) are superimposed upon the boxplots of the distributions. The descriptors with names beginning with “FG” represent descriptors calculated for the neutral molecular structures of the outermost functional groups. “X_metal” denotes the Pauling metal atom electronegativity descriptor. See section “Descriptor calculations” under the Experimental section for detailed explanations of the names of the other descriptors.

Here, it should be noted that the Gini importance measure for Random Forest [56] is known to be biased towards continuous variables and variables with multiple values, as opposed to binary variables and permutation-based importance measures are expected to be more reliable [57–58]. (Typically, the change in accuracy on the out-of-bag samples for the final, fitted Random Forest is considered [39], but the change in cross-validated balanced accuracy, including rescaling the descriptors and retuning the hyperparameters on the training set, was considered in the current work for consistency with analysis of the logistic regression model and in keeping with prior work [54].) It is possible that similar considerations affect the logistic regression coefficient magnitudes. In the current work, biases towards continuous variables could result in a failure to identify the binary variables representing different qualitative characteristic values for core shape, surface charge and purity [31], or the presence/absence of surface functional groups as important. Also, for example, elevated significance could be assigned to variables such as the Pauling metal atom electronegativity and the average primary particle size. Nonetheless, the importance of the Pauling metal atom electronegativity descriptor is confirmed based upon consideration of a conditional variable importance estimate (Figure 11) obtained using a variation on the Random Forest modelling and variable importance protocol, which can be expected to produce reliable estimates of descriptor importance [58].

The finding from these analyses (Figure 10 and Figure 11), taking into account the differential degrees of reliability discussed above, that core composition characteristics, notably the metal atom electronegativity, are most significantly related to excess lethality at 120 hpf, with limited influence of descriptors representing the primary particle size or surface characteristics is interesting. The finding that the Pauling metal atom electronegativity descriptor was of greatest significance is broadly in keeping with earlier studies that found that this descriptor could be used, along with a few other basic variables representing core composition that were also considered herein, to model the cytotoxicity of a wide variety of metal oxide ENMs [55,59]. It has been proposed that the electronegativity of the metal atom may correlate well with the electronegativity of the cation, such that a descriptor based upon the metal atom electronegativity may reflect catalytic activity due to ions release via dissolution, leading to toxicity via generation of reactive oxygen species (ROS) [60]. However, metal oxides may also result in toxicity via a differential ability to bind to the surface of zebrafish embryos or direct inhibition of enzymes by the released cations [60–61].

Further research is required here into the basis for the importance of the Pauling metal atom electronegativity in our models. We note that the fact that different oxidation states of the same metal were not considered in our dataset may also have resulted in a variable related purely to the metal atom identity being assigned elevated significance.

Our finding that surface characteristics are of negligible importance, compared to core chemical composition is at odds with some previous analyses. These analyses suggested that outermost surface features were more significant drivers of variability in biological effects than core chemical composition [28,31]. However, our finding that primary particle size is of limited significance is in keeping with earlier analyses. Previous analyses of NBI Knowledgebase data have suggested that, whilst primary particle size may not be irrelevant to observed biological effects, no consistent trends could be observed with primary particle size and/or that surface chemistry was a more significant factor driving variability in biological effects [28–29,31].

It is possible that our results regarding the limited significance of surface characteristics may be skewed by the manner in which surface chemical composition was represented using descriptors and the incomplete availability of surface charge information. (See the sections “Descriptor calculations”, “Inapplicable descriptor dummy values” and “Descriptor pre-processing” under the Experimental section for full details.) First, the presence or absence of organic surface components was represented using binary indicator variables. Their negligible significance for modelling of the 120 hpf excess lethality data could reflect biological significance arising from the nature of any surface coatings, rather than the presence or absence of any organic coating having general significance. Second, qualitative surface charge information, including “unknown” status, was represented using binary variables for each value. Their negligible significance might partly reflect the limited availability of this information. Finally, the specific chemical composition of the organic surface coatings was represented using molecular descriptors designed to capture characteristics related to intermolecular interactions, which could affect agglomeration or uptake by cells, and reactivity, which could trigger toxicity. (Dummy values, lower than the minimum of observed values, were inserted where the corresponding coating was absent, in keeping with recommended practice [45].) Here, molecular descriptors were calculated for the gas phase, neutral forms of the molecular constituents of the surface components. (This approach is similar to previous modelling studies of NBI Knowledgebase data, where, in addition to exploring the identities of the surface functional groups as variables related to biological effects, surface chemical composition was encoded using molecular descriptors computed for the gas phase molecules [28–29], with pH value-dependent ionization state reported to have been taken into account in one study [29].) In reality, these molecules are likely to be bound to the nanomaterial surface [62–64] and it is possible this binding could even alter the molecular structure [64].

#### Selection of a single descriptor Random Forest model for external validation

The finding that, out of all descriptors used for all previously analysed models, only the Pauling metal atom electronegativity could be reliably considered highly significant prompted investigation of whether an approximate model of excess lethality at 120 hpf could be based purely upon this descriptor. On pragmatic grounds, such an approximate model would also be useful as a first-step screening tool, in cases where more detailed physicochemical characterisation data were missing [65]. Here, it was found that a Random Forest model based upon this descriptor gave comparable or better cross-validation statistics to the original Random Forest and logistic regression models based upon all descriptors (Table 1). Hence, this approach was used to train a model on the entire model development dataset prior to external validation. It was found that, as expected for tree based models, the cross-validated performance statistics were unchanged if the descriptor was unscaled, hence the final model for external validation was built without scaling the Pauling metal atom electronegativity descriptor. This simplifies applications to new NMs.

#### Applicability domain

As a first approximation [66], the training set range of the Pauling metal atom electronegativity descriptor (1.12–2.05), that is, the only descriptor, was used to define an applicability domain for the selected model.

#### External validation results

As previously indicated, all of the individual cross-validation results presented herein were generated using a nested cross-validation framework, such that any selection of model parameters or descriptors was carried out independently for each cross-validation split, using the cross-validation training set alone, to avoid optimistic bias in the model performance statistics [43–44]. (The one exception to this was the use of the entire model development set to select dummy values for surface component descriptors, but this is not expected to have any influence on the results with Random Forest, and the weak importance assigned to these descriptors indicates this was not a cause of significant optimistic bias for any method.) However, it should be acknowledged that the exploration of different modelling approaches, especially the final selection of a single descriptor model based upon descriptor importance analysis performed using all 44 ENMs, might have resulted in optimistic bias.

Hence, an assessment of the performance of the single descriptor model on external data would be of value to assess this. However, limited data were available from the NBI Knowledgebase for true external validation. In total, seven metal oxide ENMs were identified, by relaxing certain selection criteria, which led to these not being included in the initial set of 44 used for model development and cross-validation. This entailed (i) allowing for the inclusion of minimally doped ENMs, (ii) allowing for the selection of ENMs with missing numerical characterisation data used as input variables to the original, multi-descriptor models, and (iii) allowing for the selection of materials tested at maximum dosage concentrations of less than 250 ppm. All of these ENMs were determined to not have a LOEL up to 250 ppm for excess lethality at 120 hpf, that is, they were considered non-toxic according to the modelled endpoint. (Of course, they may exhibit toxic effects for other endpoints.) However, the four materials tested at maximum concentrations less than 250 ppm might be false negatives.

All ENMs lay inside the applicability domain of the selected single descriptor model. All of them were correctly classified as “non-toxic”. However, given the limitations of this dataset, including the absence of toxic examples and the possibility of experimental false negatives, further studies are required to assess how predictive the selected one descriptor model is on truly external data.

Additional analyses were performed on indirectly comparable, small datasets curated from the literature and the results are reported in Supporting Information File 1. (See section “Selection of data for external validation of the final single descriptor model” under the Experimental section for further details.) However, due to the limitations of these datasets, in terms of their suitability for assessing the predictive performance of the selected model, these results are not discussed here.

#### Key contributions of this research and future directions

The work presented herein represents the first time that classification models of Nanomaterial Biological-Interactions Knowledgebase hazard data, a diverse set of ENM hazard data derived from experiments in a human safety relevant test system performed in a single laboratory, have been reported. More generally, it has demonstrated, for the first time, that diverse metal oxide ENMs, coated and uncoated with different core compositions, with and without LOEL values up to 250 ppm for lethality with respect to embryonic zebrafish during a specific time period, may be successfully distinguished using classification techniques and calculated descriptors. More specifically, it was demonstrated that ENMs causing lethality during the time period from 24 to 120 hpf (i.e., excess lethality at 120 hpf) could be successfully identified. Interestingly, it was found that comparable results could be obtained using a model based upon a single, simple descriptor: the Pauling electronegativity of the metal atom. This descriptor has previously been used to model cytotoxicity of metal oxides [55,59], and other electronegativity-based descriptors have been used to model diverse ENM data, including LC_50_ values for some metal oxide ENMs tested at different time points against embryonic zebrafish [67] and metal oxide inhibition of the zebrafish hatching enzyme [60]. However, we are unaware of any previous studies demonstrating that this simple electronegativity descriptor on its own could be used to discriminate metal oxide ENMs with differential lethal effects against embryonic zebrafish. Given the growing interest in the use of embryonic zebrafish as an initial hazard screening tool for metal oxide ENMs [24], this suggests that the very simple, single descriptor model we identified herein could be of value in providing manufacturers of ENM-enabled products with a rapid initial hazard ranking prior to synthesis and testing.

Nonetheless, we do not suggest that the toxicity of diverse ENMs towards embryonic zebrafish is purely related to this simple descriptor providing limited information about the composition of the metal oxide core. Numerous studies have shown that a variety of intrinsic and extrinsic characteristics may influence the level of hazard associated with ENMs in general [65] and for metal oxides applied to embryonic zebrafish in particular [24].

Future studies should examine whether significantly better classification of the lethality of metal oxide ENMs applied to embryonic zebrafish could be obtained if more sophisticated descriptors of the ENM surfaces were employed. This could entail calculating the descriptors at biologically relevant pH values [29], as well as constructing physically realistic representations of the surface structures of coated and uncoated nanomaterials, rather than calculating descriptors for organic surface components as free molecules and substituting dummy values in the absence of such components. Of course, approximate representations of the ENM surface structures would need to be constructed in the absence of full information, for example, surface ligand density. More generally, various recent publications have proposed sophisticated calculated descriptors that might be suitable for improved modelling of these data [68–71]. In addition, the possibility of improving the results by replacing the Pauling electronegativity values with those from a very recently published electronegativity scale [72] should be explored.

This work also found that data augmentation using the “noised training set replication” approach of Cortes-Ciriano and Bender [36] or a novel approach (“weighted alternative samples”), which was analogous to the approach previously applied by Kim et al. [35], failed to clearly improve performance. To the best of our knowledge, this represents the first time that these data augmentation approaches have been investigated as means of addressing the recognised problem of modelling the typically small datasets available for nano-QSAR modelling [20,33–34]. Indeed, to the best of our knowledge, this represents one of the few times that any data augmentation strategies have been investigated in nano-QSAR studies. Yan and co-workers [73], in a recent study modelling the biological activity of ENMs for which image-based representations of their structural data could be generated, considered the inclusion of multiple different image-based representations (“screenshot from multiviews”) of the same ENM structures in the training set. It would be interesting to see if this approach could be adapted to model the data reported herein, albeit the representations of ENM structure they used were generated based upon the surface ligand density, which is not reported for ENMs in the NBI Knowledgebase. In addition, Subramanian et al. [74] recently reported the use of the SMOTE algorithm [75] to address class imbalance. Whilst not formally described as data augmentation, the generation of synthetic members of the minority class by SMOTE [75] is clearly analogous to the “noised training set replication” paradigm [36] and the possibility of using this to improve modelling of the dataset reported herein should be explored in future work. Finally, it is possible that the poor performance of the “noised training set replication” paradigm here, in contrast to previous studies [36], reflects the descriptors involved. Hence, future studies should investigate whether adding different levels of noise to different kinds of descriptors, such as the novel descriptors discussed above, would offer improved performance.

Separately, one particularly interesting finding of this work was that classifying ENMs according to their LOEL for lethality up to 24 hpf was not clearly possible, whereas it does appear possible to build models identifying ENMs exhibiting lethal effects at 250 ppm or below in the timeframe 24 to 120 hpf (i.e., excess lethality at 120 hpf). This represents a notable challenge, as mortality at 24 hpf indicates a stronger acute toxic response and, ideally, it would be possible to classify ENMs based upon their cumulative lethal response at 120 hpf as well as identify those causing mortality at 24 hpf. (One approach to doing so would be to combine the models developed here for excess lethality at 120 hpf and improved models for mortality at 24 hpf.) It is possible that our findings reflect the fact that toxicity up to 24 hpf requires dermal penetration to reach the site of biological action, since swallowing does not start until around 72 hpf [31]. Hence, descriptors better describing dermal penetration may allow for ENMs exhibiting lethal effects up to 24 hpf to be better identified. Of course, other possible interpretations of our results could be different toxicological modes of action operating in the period 24 to 120 hpf, as embryonic cells differentiate, as well as the possibility of delayed mortality induced by the initial exposure prior to 24 hpf. For example, if toxicity is caused by disrupting the function of hatching enzymes [60–61], this could result in embryo mortality via starvation [61]. However, as hatching normally happens within 72 hpf [76], the significance of disrupting hatching may not manifest until after 24 hpf.

However, one further factor that may affect the findings herein is the fact that the LOELs used to define the modelled categories were assigned based upon mass-based concentrations. The issue of dosimetry, that is, the most appropriate dose units to characterise the differential hazard of different ENMs, remains an area under active consideration in the nanotoxicology community [65,77–78]. Whilst applied surface area or particle number doses might be more appropriate [78], albeit this is not accepted in all studies [77], computing these requires either specific surface area measurements, or estimates to be made based upon additional physicochemical characterization data, such as particle shape, agglomerate size and/or particle density [77–79]. However, these physicochemical characterization data are not reported for all studied ENMs, including for all ENMs reported in the Nanomaterial Biological-Interactions Knowledgebase. One alternative possibility might be to examine how the results reported herein would change if the LOEL data were expressed in terms of pseudo-moles [18,30,67], which could be estimated, to a first approximation, by dividing the mass-based dose by the relative mass of the formula unit of the core material, for example, ZnO.

Whilst care was taken to ensure that all individual cross-validation results were not optimistically biased, by carrying out the selection of all hyperparameters independently via internal cross-validation on each cross-validation training set, the exploration of different modelling results and, in particular, the identification of the Pauling electronegativity descriptor for the final, selected model, using all data employed for cross-validation, may have introduced optimistic bias. A partial external validation on the limited available data, not considered for the initial analyses and comparisons of different approaches, suggested the model was correctly able to identify them as not having a LOEL value (for excess lethality at 120 hpf) up to a test concentration of 250 ppm. Nonetheless, future experimental studies would help to confirm the true external predictivity of the model. These studies should generate comparable data to those modelled in the current work, including similar tested doses, exposure and sampling time periods following the application of metal oxide ENMs to embryonic zebrafish.

## Conclusion

This work demonstrated, for the first time, that structurally diverse (coated and uncoated with different core compositions) metal oxide nanomaterials can potentially be classified according to whether they have a lowest observed effect level, up to 250 ppm, for excess lethality against embryonic zebrafish measured in the period from 24 to 120 hpf. However, the fact that it was not clearly possible to identify nanomaterials with a lowest observed effect level, up to 250 ppm, for lethality in the period from the onset of exposure up to 24 hpf might be related to the need to consider more appropriate descriptors to represent different modes of exposure to the site of biological action.

The initial models were developed using a variety of experimental and calculated descriptors related to physicochemical characteristics, including approximate representations of the core composition and, where applicable, organic surface ligands. (Care was taken, using a nested cross-validation protocol, to minimise optimistic bias in the estimated performance of these models.) However, interestingly, it was found that a predictive model with comparable or better results might be obtained using a single, simple descriptor related to core chemical composition: the Pauling electronegativity of the metal atom.

The selected single descriptor model could be used for an initial hazard ranking, even prior to synthesis, by manufacturers of nanomaterial enabled products, as part of a “safe by design” paradigm. However, the fact that model validation was primarily performed using cross-validation, on data used to identify the significance of the selected descriptor, means that future experimental studies are required to generate additional, comparable data for robust, truly external validation of this model.

Since the dataset modelled herein was relatively small (44 nanomaterials), albeit still larger than the datasets used in many previous nano-QSAR studies, the possibility of improving the predictive performance using two data augmentation techniques was explored. To the best of our knowledge, this is the first time that the use of either of these techniques to improve modelling results has been investigated in the nano-QSAR literature. However, in the current context, no improvement in performance was observed. This issue should be investigated more widely in the nano-QSAR community, as should whether the models reported herein could be improved via using more sophisticated descriptors reported in the recent literature.

## Experimental

### Computational details

All code required to reproduce the results herein has been made available on Zenodo [80] under the terms of the Open Source GNU Public License (version 3). The code archive is divided into two sub-folders: (1) \BioRima_calc_UoL.final\ and (2) \BioRima_MarcheseRobinsonEtAlExtraCalc\. The former sub-folder corresponds to the scripts used to carry out all calculations performed to process the data from the Nanomaterial Biological-Interactions Knowledgebase, to derive the model development set, cross-validate the multiple descriptor Random Forest models with and without data augmentation, and perform the analysis of those initial results. The latter sub-folder corresponds to the scripts used to perform all additional calculations reported in this paper. Supporting Information File 1 includes a step-by-step guide to reproducing all results using these scripts (“How to Reproduce Our Results”). This includes details of how to install the exact versions of all software dependencies employed for all calculations, including by making use of the environment files, included in the code archive, required to create the conda environments used to run the calculations performed using different Python 3 and R scripts.

Both sets of calculations were performed on machines running a Windows operating system (64-bit architecture). The details of the machine used to perform the first set of calculations were as follows: Windows 7 Enterprise Service Pack 1 (64-bit). Processor: Intel(R) Core™ i5-6300U CPU @ 2.40 GHz. Installed memory (RAM): 8 GB (7.41 GB usable). The details of the machine used to perform the second set of calculations were as follows: Windows 10 Home (64-bit). Processor: AMD Ryzen 5 3500 U with Radeon Vega Mobile Gfx 2.10 GHz. Installed memory (RAM): 8 GB (5.94 GB usable).

### Selection of data for model development purposes

All data modelled herein were obtained using common experimental protocols, with some differences in the details (e.g., small changes in medium composition and temperature for the biological assay, and different physicochemical characterization techniques). All biological data were generated in a single laboratory (Harper Laboratory, Oregon State University). A detailed description of the experimental protocol used to generate the biological data was previously reported [28] and a summary, including the most relevant details for the current work, is provided below.

Each nanomaterial was exposed to embryonic zebrafish (*Danio Rerio*) at a range of different dosage concentrations. The number of dosage concentrations and the maximum concentration varied somewhat between different nanomaterials. Each study of a given nanomaterial also used a zero-dose control group, which allowed for an assessment of statistically significant differences due to different dosage concentrations with respect to the absence of nanomaterial exposure (see below). For each dosage concentration, including the zero-dose control, a group of between 24 and 48 embryonic zebrafish were exposed via continuous exposure in fish water medium (commonly reported as pH 7.1–7.2 and *T* = 23–27 °C), starting at 7–8 hours post-fertilization (hpf). At 24 and 120 hpf, non-invasive observations were made of the number of embryonic zebrafish exhibiting signs of both lethal (mortality) and non-lethal (see below) abnormalities. Counts of the numbers of embryos exhibiting and not exhibiting a given abnormality were recorded. For all endpoints other than mortality at 24 hpf, these counts were based upon the number of fish that survived at 24 hpf. Hence, the mortality counts at 120 hpf reflect excess lethality in the period of 24–120 hpf.

In addition to these biological data, qualitative and quantitative characterization data were reported for various intrinsic and extrinsic physicochemical characteristics. These characterization data were reported to varying extents for different nanomaterials. All data and experimental metadata were extracted from the data records, exported from the Nanomaterial Biological-Interactions (NBI) Knowledgebase [26], and reported in the NanoHub online repository [81] as supplementary material for Karcher and co-workers [31]. Each set of data on a given nanomaterial (including provenance and synthesis metadata, along with physicochemical characterisation and biological data plus metadata) corresponded to a distinct data file, associated with a unique NBI Material Identifier.

These data files were processed using an automated workflow, implemented using Python 3 code, to select a subset of data that were compliant with the following selection criteria: (1) the nanomaterial was described as “metal oxide”; (2) the “Core Atomic Composition” and “Particle Descriptor” fields did not indicate the presence of multiple metal or, in one case, metalloid (silicon) cations (i.e., doped or mixed oxidation state metal oxides were rejected); (3) the material was tested at a maximum dose of 250 ppm (i.e., any materials tested at lower or higher maximum doses were rejected); (4) the following fields, reporting qualitative and quantitative physicochemical characterization data for which relationships with biological data were previously explored by Karcher et al. [31], were not empty (i.e., “Surface Charge: (positive, negative, neutral)”, ”Primary Particle Size: Avg. (nm)”, ”Outermost Surface Functional Groups”, ”Core Atomic Composition”, ”Shell Composition”, “Purity”, ”Core Shape”, ”Core Structure”).

In addition, prior to filtering the nanomaterial data records, processing of each nanomaterial data record included the derivation of statistically significant lowest observed effect level (LOEL) values for lethality at 24 hpf and excess lethality in the period from 24 to 120 hpf based upon mortality observations at 24 and 120 hpf, respectively. Additional updates were performed prior to the filtering steps described above: (a) in keeping with Karcher et al. [31], missing “Primary Particle Size: Avg. (nm)” entries were replaced with the corresponding “Primary Particle Size: Max (nm)” values, if the corresponding “Primary Particle Size: Min. (nm)” entry was also blank, which could reflect inconsistent population of the data fields; (b) inconsistently populated fields, where they were identified, were normalized (e.g., "Exposure Organism Average Weight (mg)" values of “1 mg” were replaced with 1.0 and “na” values were replaced with “none”); (c) prior to computing the LOEL values, any nanomaterial IDs that were not associated with a zero-dose control group were removed.

Dataset files derived from the NBI Knowledgebase data files, at various stages of processing and filtering, are made available in Supporting Information File 2.

### Determination of statistically significant LOEL values

Lowest observed effect level (LOEL) values [32] were independently assigned for each endpoint. They were derived from data showing the number of tested embryonic zebrafish at different exposure concentrations, that is, dose levels, including zero-dose control groups, for which the relevant biological effect, for example, mortality, was observed and not observed. For all endpoints other than mortality at 24 hpf, the numbers of embryonic zebrafish for which the effect was observed or not observed in the zero-dose control group or at a specific exposure concentration were evaluated with respect to the total number of embryonic zebrafish left alive after 24 hpf. This means that the mortality data reported at 120 hpf actually corresponds to excess lethality, that is, lethality occurring between 24 and 120 hpf, in excess of any lethality that occurred up to 24 hpf. By modelling excess lethality at 120 hpf, rather than the total lethality at 120 hpf, this reduced the potential mixing of mortality occurring due to different exposure routes prior to and following 24 hpf [31].

Fisher’s exact test [82], as implemented in the Python module *scipy.stats* [83], was used to assess the statistical significance, at the 5% level, of an increase in the effect, compared to the zero-dose control group, at a given dose. After each dose was assessed for statistical significance, the tested concentrations were then ranked from highest to lowest. This list was descended and the highest tested concentration was checked for a statistically significant positive association with the endpoint. If this was detected, the LOEL value was initially assigned to this highest tested concentration. If the next highest concentration was also associated with a statistically significant finding, the LOEL was reassigned to this concentration. This downwards adjustment of the LOEL was continued until a concentration was encountered that was not associated with a statistically significant result. If the highest tested concentration was not associated with a statistically significant result, no LOEL was assigned, irrespective of whether lower dose levels were determined to be individually statistically significantly associated with the effect.

This protocol was designed to ensure that LOEL values were only assigned where a statistically valid dose response curve was observed, showing an increase in biological response with increasing dose as would generally be expected, and reducing the chance of spurious LOELs being detected due to a statistical fluke, in keeping with common practice in toxicology [32]. However, this procedure would still detect a LOEL at the highest dose if the highest dose was associated with a statistically significant response, even if a statistically significant response was observed at a lower dose and not at intermediate higher doses, or no significant responses were observed at other doses. Moreover, if a statistically valid dose response curve for excess lethality at 120 hpf occurred at lower doses but mortality at 24 hpf was sufficient to kill all embryos at higher doses, this protocol could fail to detect a valid LOEL for excess lethality at 120 hpf, that is, there would be no surviving embryos left to die in the period from 24 to 120 hpf at the higher doses. In practice, only one such apparent “false negative”, that is, an ENM wrongly considered “non-toxic” due to the absence of a LOEL being detected up to the maximum tested concentration (see below), was present in the model development dataset used in the current study (NBI Material Identifier = 214). In addition, there were only two other ENMs in the model development dataset for which a LOEL for excess lethality at 120 hpf was not detected because statistically significant responses were observed at lower doses but not at higher doses and this was not due to no embryos surviving beyond 24 hpf (NBI Material Identifiers = 195 and 136). Whilst it might be argued that the latter could represent cases of non-monotonic dose response, which might arise with certain toxicological mechanisms [84], this would not generally be expected and could represent statistical flukes. Indeed, applying the Benjamini and Yekutieli multiple testing correction [85], treating the *p*-values for excess lethality at 120 hpf at each tested concentration of a specific ENM as a separate family, results in the corrected *p*-values for these latter ENMs (NBI Material Identifiers = 195 and 136) no longer being statistically significant at the 5% level.

### Meaning of “inactive vs active” and “non-toxic vs toxic” classifications

Since the data were selected such that the highest tested dose concentration was 250 ppm (mass-based concentration units), this means that any nanomaterials without a LOEL value less than or equal to 250 ppm, for the endpoint under consideration, were considered inactive, in the case of sub-lethal endpoints, or non-toxic, in the case of the unambiguously adverse endpoints of lethality (mortality) or excess lethality at 24 and 120 hpf respectively [32]. Otherwise, the nanomaterials were considered active or toxic respectively. However, since only the embryos that survived beyond 24 hpf, for either the non-exposed (zero dose) or exposed groups, were used to determine the LOEL values for the other endpoints, this means that the labels assigned for the 120 hpf mortality endpoint do not account for biological activity observed up to 24 hpf. Hence, the binary classification endpoint values at 120 hpf should be interpreted as excess lethality for the mortality endpoint.

#### Endpoint abbreviations

The abbreviations are explained in Table 2. Further details regarding these endpoints are provided in Harper and co-workers [28].

**Table 2:** Abbreviations used to label the different endpoints corresponding to different biological changes observed at either 24 and/or 120 hours post-fertilisation.

Abbreviation	Expanded description	Lethal or sub-lethal?

M	mortality	lethal
DP	delayed developmental progression	sub-lethal
SM	lack of spontaneous movement	sub-lethal
N	notochord malformation	sub-lethal
J	jaw malformation	sub-lethal
Y	yolk sac edema	sub-lethal
A	curved axis	sub-lethal
E	eye malformation	sub-lethal
Sn	snout malformation	sub-lethal
O	otic vesicle malformation	sub-lethal
H	heart malformation	sub-lethal
B	brain malformation	sub-lethal
So	somite malformation	sub-lethal
PF	pectoral fin malformation	sub-lethal
CF	caudal fin malformation	sub-lethal
P	atypical pigmentation	sub-lethal
C	occluded circulation	sub-lethal
T	trunk malformation	sub-lethal
SB	atypical swim bladder inflation	sub-lethal
TR	lack of touch response	sub-lethal

#### Descriptor calculations

A variety of numerical descriptors were derived from a set of qualitative and quantitative physicochemical characteristics selected from those examined for a link with biological activity by Karcher and co-workers [31]. Two characteristics were rejected on the grounds that they were either constant (material type) for the modelled data, that is, all dataset entries were labelled as metal oxides, or the characteristic only took one value other than “unknown” (core structure), making them uninformative.

The quantitative characteristic, treated directly as a descriptor, was average primary particle size. (In keeping with Karcher et al. [31] the reported maximum size was used in lieu of a missing average size, if the minimum value was also missing. This might reflect inconsistent recording of data.) The qualitative characteristics were as follows: surface charge (positive, negative, neutral), purity, core shape, core atomic composition and information regarding the chemical composition of the shell and/or outermost surface functional groups (if applicable). With the exception of the core, shell and outermost surface chemical composition descriptions, these characteristics were one-hot-key encoded. This entailed creating, for each characteristic, a separate numeric descriptor for each of the reported qualitative values (including some “unknown” values). These descriptors took the values one or zero if the value for the characteristic corresponded or did not correspond to the value used to define the descriptor, respectively.

The chemical composition of the core was approximately represented using the following descriptors, in keeping with a previous study, which found these to be useful for modelling nanosized metal oxide cytotoxicity data [55]: Pauling metal atom electronegativity, number of metal cations in the oxide, number of oxygen anions in the oxide and charge of metal cation in the oxide. The chemical composition of the shell and outermost surface functional groups, where applicable, was approximately represented via computing a set of molecular descriptors from SMILES strings representing the neutral forms of the free molecules. (These SMILES are reported in the code archive made available on Zenodo [80].) These molecular descriptors were as follows: (1) HOMO–LUMO gap; (2) HOMO energy; (3) LUMO energy; (4) McGowan volume; (5) Wildman–Crippen molar refractivity (SMR); (6) Labute’s approximate surface area (LabuteASA); (7) topological polar surface area (TPSA) and (8) approximations of the Absolv descriptors corresponding to the solvation parameters originally proposed by Abraham [86], not including the directly estimated McGowan volume. These descriptors were selected to reflect the potential for reactivity, in the case of the descriptors related to the HOMO and LUMO energies, as well as effects on nanomaterial surface interactions [87] and were informed by a previous local nano-QSAR study of the effect of surface composition and concentration on the biological response of coated gold nanoparticles [28].

The electronic structure descriptors (HOMO–LUMO gap as well as HOMO and LUMO energy) were computed using the MOPAC2016 implementation of the semiempirical quantum chemical method PM7 [88]. These calculations were performed on estimates of the lowest-energy conformer for the standardised structure of the molecule represented by the available SMILES string, where an initial conformer was generated using the ETKDG algorithm [89], followed by a global conformer search using the MMFF94 force field [90]. The approximated Abraham solvation parameters were as follows: R2 (E - solute excess molar refraction, representing the potential for dispersion interactions), Pi2 (S - solute dipolarity-polarizability, representing the potential for dipole-induced dipole interactions), BetaH2 (B - solute H-bond total basicity, representing H-bond acceptor ability) and AlphaH2 (A - solute H-bond total acidity, representing H-bond donor ability). These were calculated via building a Support Vector Regression model [91], using an extended connectivity fingerprint [92] and a Tanimoto kernel [93], trained on a set of previously reported Absolv calculations on a molecular dataset [94]. The remaining descriptors were computed using the RDKit [95] or Mordred [96].

When the nanomaterial in question had no shell and/or organic outermost surface functional groups, that is, was uncoated, dummy values were used for all of the corresponding descriptors (see below). Finally, the presence or absence of shell or organic outermost surface functional groups was explicitly encoded using two descriptors taking the values one (present) or zero (absent).

The full set of descriptor names, or the descriptor name prefixes for the one-hot-key encoded qualitative variables, as reported in the model development dataset file provided in Supporting Information File 2, is summarized in Table 3. As explained below, the descriptors corresponding to the molecular structures of the outermost surface functional groups or shell were set to dummy values when these components were not present. In addition, as also explained below, the one-hot-key encoded qualitative descriptors and the binary descriptors corresponding to the presence or absence of outermost surface functional groups or a shell were not perturbed as part of data augmentation, as these can only take discrete values of one or zero.

**Table 3:** Summary of all descriptor names, or prefixes for the one-hot-key encoded qualitative variables, reported in Supporting Information File 2, along with a description of how they were derived.

Descriptor name or prefix for qualitative variables	Quantitative or qualitative variable?	Description	Source

Surface Charge: (positive, negative, neutral)	qualitative	qualitative physicochemical characterisation data reported in the NBI Knowledgebase	characterisation data
Primary Particle Size: Avg. (nm)	quantitative	quantitative physicochemical characterisation data reported in the NBI Knowledgebase	characterisation data
Outermost Surface Functional Groups: Present?	quantitative [binary]	binary variable (values = 1 or 0) based upon whether any outermost surface functional groups are reported in the NBI Knowledgebase	characterisation data
Shell: Present?	quantitative [binary]	binary variable (values = 1 or 0) based upon whether any shell is reported in the NBI Knowledgebase	characterisation data
purity	qualitative	physicochemical characterisation data reported in the NBI Knowledgebase	characterisation data
core Shape	qualitative	physicochemical characterisation data reported in the NBI Knowledgebase	characterisation data
Pauling metal atom electronegativity	quantitative	electronegativity (Pauling scale) for the metal atom corresponding to the cation in the metal oxide core	calculated value reported in the literature [55] or by the Royal Society of Chemistry [97]
number of metal cations in the oxide	quantitative	number of metal cations in the formula unit of the metal oxide core	defined by the formula unit
number of oxygen anions in the oxide	quantitative	number of oxygen anions in the formula unit of the metal oxide core	defined by the formula unit
charge of metal cation in the oxide	quantitative	formal oxidation state of the metal in the metal oxide core	defined by the formula unit
FG: GAP (eV)	quantitative	HOMO–LUMO gap for the lowest energy conformer of the standardised molecular structure of the outermost surface functional groups (or a dummy value if the ENM does not possess this)	MOPAC2016 (PM7) [conformer generation performed using RDKit]
FG: HOMO energy (eV)	quantitative	HOMO energy for the lowest energy conformer of the standardised molecular structure of the outermost surface functional groups (or a dummy value if the ENM does not possess this)	MOPAC2016 (PM7) [conformer generation performed using RDKit]
FG: LUMO energy (eV)	quantitative	LUMO energy for the lowest energy conformer of the standardised molecular structure of the outermost surface functional groups (or a dummy value if the ENM does not possess this)	MOPAC2016 (PM7) [conformer generation performed using RDKit]
FG: McGowanVolume	quantitative	McGowan volume for the molecular structure of the outermost surface functional groups (or a dummy value if the ENM does not possess this)	Mordred (1.2.0)
FG: SMR	quantitative	Wildman–Crippen molar refractivity descriptor for the molecular structure of the outermost surface functional groups (or a dummy value if the ENM does not possess this)	Mordred (1.2.0) [RDKit wrapper]
FG: LabuteASA	quantitative	Labute’s approximate surface area descriptor for the molecular structure of the outermost surface functional groups (or a dummy value if the ENM does not possess this)	Mordred (1.2.0) [RDKit wrapper]
FG: TPSA	quantitative	topological polar surface area for the molecular structure of the outermost surface functional groups (or a dummy value if the ENM does not possess this)	RDKit (2019.03.3.0)
FG: approx_D_R2_E_	quantitative	approximation of the Absolv descriptor R2 (E - solute excess molar refraction, representing the potential for dispersion interactions) for the molecular structure of the outermost surface functional groups (or a dummy value if the ENM does not possess this)	Support Vector Regression model trained on Absolv calculations for a molecular dataset
FG: approx_D_Pi2_S_	quantitative	approximation of the Absolv descriptor Pi2 (S - solute dipolarity-polarizability, representing the potential for dipole-induced dipole interactions) for the molecular structure of the outermost surface functional groups (or a dummy value if the ENM does not possess this)	Support Vector Regression model trained on Absolv calculations for a molecular dataset
FG: approx_D_BetaH2_B_	quantitative	Approximation of the Absolv descriptor BetaH2 (B - solute H-bond total basicity, representing H-bond acceptor ability) for the molecular structure of the outermost surface functional groups (or a dummy value if the ENM does not possess this)	Support Vector Regression model trained on Absolv calculations for a molecular dataset
FG: approx_D_AlphaH2_A_	quantitative	approximation of the Absolv descriptor AlphaH2 (A - solute H-bond total acidity, representing H-bond donor ability) for the molecular structure of the outermost surface functional groups (or a dummy value if the ENM does not possess this)	Support Vector Regression model trained on Absolv calculations for a molecular dataset
Shell: GAP (eV)	quantitative	HOMO–LUMO gap for the lowest energy conformer of the standardised molecular structure of the shell (or a dummy value if the ENM does not possess this)	MOPAC2016 (PM7) [conformer generation performed using RDKit]
Shell: HOMO energy (eV)	quantitative	HOMO energy for the lowest energy conformer of the standardised molecular structure of the shell (or a dummy value if the ENM does not possess this)	MOPAC2016 (PM7) [conformer generation performed using RDKit]
Shell: LUMO energy (eV)	quantitative	LUMO energy for the lowest energy conformer of the standardised molecular structure of the shell (or a dummy value if the ENM does not possess this)	MOPAC2016 (PM7) [conformer generation performed using RDKit]
Shell: McGowanVolume	quantitative	McGowan volume for the molecular structure of the shell (or a dummy value if the ENM does not possess this)	Mordred (1.2.0)
Shell: SMR	quantitative	Wildman–Crippen molar refractivity descriptor for the molecular structure of the shell (or a dummy value if the ENM does not possess this)	Mordred (1.2.0) [RDKit wrapper]
Shell: LabuteASA	quantitative	Labute’s approximate surface area descriptor for the molecular structure of the shell (or a dummy value if the ENM does not possess this)	Mordred (1.2.0) [RDKit wrapper]
Shell: TPSA	quantitative	topological polar surface area for the molecular structure of the shell (or a dummy value if the ENM does not possess this)	RDKit (2019.03.3.0)
Shell: approx_D_R2_E_	quantitative	approximation of the Absolv descriptor R2 (E - solute excess molar refraction, representing the potential for dispersion interactions) for the molecular structure of the shell (or a dummy value if the ENM does not possess this)	Support Vector Regression model trained on Absolv calculations for a molecular dataset
Shell: approx_D_Pi2_S_	quantitative	approximation of the Absolv descriptor Pi2 (S - solute dipolarity-polarizability, representing the potential for dipole-induced dipole interactions) for the molecular structure of the shell (or a dummy value if the ENM does not possess this)	Support Vector Regression model trained on Absolv calculations for a molecular dataset
Shell: approx_D_BetaH2_B_	quantitative	approximation of the Absolv descriptor BetaH2 (B - solute H-bond total basicity, representing H-bond acceptor ability) for the molecular structure of the shell (or a dummy value if the ENM does not possess this)	Support Vector Regression model trained on Absolv calculations for a molecular dataset
Shell: approx_D_AlphaH2_A_	quantitative	approximation of the Absolv descriptor AlphaH2 (A - solute H-bond total acidity, representing H-bond donor ability) for the molecular structure of the shell (or a dummy value if the ENM does not possess this)	Support Vector Regression model trained on Absolv calculations for a molecular dataset

#### Inapplicable descriptor dummy values

Where a nanomaterial did not possess a shell or organic outermost surface functional groups, the corresponding molecular descriptors were set to dummy values chosen to lie below the minimum of the values calculated where the corresponding component was present. (This is in keeping with recommendations in the recent literature for treating data that “does not exist in principle” for the purpose of modelling studies [45].) The choice of descriptor specific dummy values was made to avoid compressing the true values into a small numerical range during descriptor scaling (see below), which might lead to problems in practice with some modelling methods. More specifically, the dummy value for the *i*-th descriptor (*D*^dummy,^*^i^*) was computed according to Equation 1, where *D*^min,^*^i^* is the minimum of the values calculated when the corresponding component was present, and *A^i^* is an adjustment factor calculated, by default, according to Equation 2, where *D*^max,^*^i^* is the maximum of the values calculated when the corresponding component was present, unless this would give a value of zero. Otherwise, *A^i^* is calculated according to Equation 3, unless this would give a value of zero, in which case *A^i^* is set to one. Here, *D*^max,^*^i^* and *D*^min,^*^i^* were calculated with respect to the entire model development set prior to any cross-validation, albeit subsequent descriptor pre-processing was carried out independently using the distribution of values for each outer cross-validation training set (see below).


[1] $$D^{\text{dummy,}i}=D^{\text{min,}i}-A^{i}$$

[2] $$A^{i}=2\left(D^{\text{max,}i}-D^{\text{min,}i}\right)$$

[3] $$A^{i}=\left|D^{\text{min,}i}\right|$$

The same principle was used to derive dummy values for other numeric ENM descriptors when ChEMBL molecular toxicity data were treated as pseudo-ENM data samples in one of the explored data augmentation techniques. In addition, for the qualitative ENM descriptors, such as core shape and purity, a fixed dummy label was assigned prior to one-hot-key encoding.

#### Descriptor pre-processing

Subsequent to descriptor calculations and the assignment of inapplicable descriptor dummy values, as described above, the descriptor values were pre-processed. Pre-processing involved converting the qualitative characteristics into numeric descriptors, with values of zero or one, using one-hot-key encoding (see above). In addition, the original numeric descriptors were mean centred, by subtracting the mean, and scaled to unit variance. These operations were carried out independently for each of the outer cross-validation training sets (see below).

For the externally validated single descriptor Random Forest model, no such descriptor pre-processing was performed, as scaling and mean centring should not affect the distribution of class labels with respect to numerical descriptors and, hence, should not affect the results obtained with tree-based models.

#### Cross-validation scheme for estimating model performance and model selection

To minimise model selection bias, double or nested cross-validation [43], sometimes referred to as “external” cross-validation [44], was employed. This means that, for a given machine learning algorithm, model hyperparameters were tuned independently for each outer cross-validation training set, with overall model performance estimated on the remaining outer fold, by determining the combination that maximised balanced accuracy estimated using an inner cross-validation split of this outer cross-validation training set. For both the outer and the inner folds 5-CV was employed.

For modelling excess lethality at 120 hpf, stratified cross-validation was employed for both the outer and inner folds. For modelling lethality at 24 hpf, after initially observing substantially worse results with Random Forest using the same nested cross-validation scheme, the outer folds were set to those selected based upon stratified cross-validation for the 120 hpf endpoint, to ensure a direct comparison was being made.

In order to better simulate the performance of the model on an external test set, for which the model would be fixed based purely upon the endpoint and descriptor values for the training set, all descriptor pre-processing was performed independently using each outer cross-validation training set. However, it should be acknowledged that the descriptor specific inapplicable descriptor dummy values (see above) were selected based upon the range of true descriptor values calculated for the entire model development set. Nonetheless, in the case of Random Forest, this should not affect the results, as the precise value of these dummy values would not affect the training set class distributions with respect to the corresponding descriptors, given that the dummy values would always lie below the true descriptor values for the training set, whether selected independently for each outer cross-validation training set or not. Hence, the split points selected for the decision trees in the forest would be unaffected.

The consideration of different modelling algorithms and the exploration of data augmentation approaches, including the exploration of different options for each paradigm as fully reported in the Results and Discussion section, could also be a source of optimistic model selection bias. However, the fact that only two modelling algorithms were considered and data augmentation never led to statistically significant improvements in model performance suggests that the results for the default modelling approaches, employing multiple descriptors, are unlikely to be optimistically biased via these investigations.

Nonetheless, the selection of the Pauling metal atom electronegativity for the single descriptor model was based upon performance metrics that considered model performance in the outer folds, hence there is a clear risk of optimistic bias in the reported cross-validation results for this model.

#### Machine Learning algorithms and tuned hyperparameters

The two machine learning algorithms employed herein were the SciKit-Learn [37] variants of Random Forest [38,40,46] and logistic regression [41–42,98]. For Random Forest, in contrast to the majority voting approach originally proposed by Breiman [38], the proportion of training set instances belonging to “class 1” (i.e., the “toxic” class in the current context) in the leaf node to which a new instance (i.e., nanomaterial in the current context) is assigned is averaged across all trees in the forest to generate the final score for “class 1”. In logistic regression, the score generated by the model is the estimated probability of “class 1”. In both cases, if the score for a new instance was greater than 0.5, this was interpreted as a prediction of “class 1” (i.e., the “toxic” class).

For logistic regression, the primal formulation was employed in combination with the liblinear solver [98]. In addition, the following sets of hyperparameter values were explored in combination via a grid search strategy: (1) *C* (inverse of regularization strength) = {1.0,10.0,100.0,1000.0}, (2) penalty = {l2, l1}. For Random Forest, the following sets of hyperparameter values were also explored in combination via a grid search strategy: (1) number of trees = {501,2001}, (2) number of descriptors randomly selected for evaluation at each split point = {sqrt(*D*), *D*/10, 2*D*/10, 3*D*/10, 4*D*/10, 5*D*/10, 6*D*/10, 7*D*/10, 8*D*/10, 9*D*/10, *D*}, where *D* is the total number of calculated descriptors, albeit this value was necessarily just set to one for the single descriptor model, (3) whether “balanced” weighting [46] was applied, when computing the reduction in Gini impurity used to choose the descriptor and split point for splitting each node [56], to avoid biasing the trees towards correctly classifying members of the majority class at the expense of performance for the minority class.

Additional hyperparameters related to data augmentation were also selected based upon the training sets of the validated models.

#### Data augmentation

For both data augmentation paradigms, the training sets used to build the validated models were augmented prior to carrying out the inner level of cross-validation on the updated training set to select other model hyperparameters. Each outer cross-validation training set was augmented independently.

For the first paradigm (“noised training set replication”), the perturbed copies of the training set added to the original training set included descriptors modified by adding random perturbations selected from a Gaussian (normal) distribution with a mean of zero and standard deviation of sigma, where sigma was treated as a tunable hyperparameter selected from the following range of values: {0.05, 0.1, 0.2, 0.3, 0.4, 0.5, 0.6, 0.8, 1.2, 1.4, 1.8}. (The one-hot-key encoded descriptors and the binary descriptors corresponding to the presence or absence of outermost surface functional groups or a shell were not perturbed, as these can only take discrete values of one or zero.) These values of sigma were based upon literature precedence [36]. Each value of sigma was considered for building a model. After optimising the machine learning algorithm hyperparameters using cross-validation on the training set, the model built using the value of sigma that maximised the training set cross-validated balanced accuracy obtained with the selected algorithm hyperparameters was selected. This process was repeated for each number of perturbed training set replicates investigated.

For the second paradigm (“weighted alternative samples”), when the samples added to the original training set were based upon molecular data selected from ChEMBL, the similarity weighting applied to each of the added samples was treated as a tunable hyperparameter selected from the following range of values: {0.1, 0.2, 0.3, 0.4, 0.5, 0.6, 0.7, 0.8, 0.9}. Again, each of these values was considered for building a model. After optimising the machine learning algorithm hyperparameters using cross-validation on the training set, the model built using the similarity weighting that maximised the training set cross-validated balanced accuracy obtained with the selected algorithm hyperparameters was selected.

Otherwise, when the samples corresponded to the same samples as the original training set, save for the fact that the endpoint values were substituted with the active (= 1) vs inactive values (= 0) for one of the sub-lethal endpoints, the similarity weighting was fixed to a value reflecting the correlation between the modelled lethal endpoint and the sub-lethal endpoint. Specifically, for all samples in the original training set, the MCC was computed between the lethal and sub-lethal endpoint. If this was positive, the similarity weighting applied to each of the added samples was set to this value. Otherwise, the similarity weighting was set to zero. In addition, if there were no active samples for a particular sub-lethal endpoint, across the entire set of NBI Knowledgebase entries for which LOEL value estimation was performed, that sub-lethal endpoint was ignored.

#### Statistical significance of cross-validation results

The outer cross-validation results obtained with the Random Forest models following the use of each data augmentation scenario were compared to the corresponding result obtained without data augmentation. The results were compared in terms of the mean balanced accuracy, MCC and AUC. Each comparison was assessed for a statistically significant increase in the result with data augmentation compared to the corresponding result without data augmentation. This assessment was based upon analysing the paired values for corresponding cross-validation folds using an exact paired permutation test [99]. This test was used to compute approximate one-tail *p*-values. Since this involved a significant number of pairwise comparisons, all of these *p*-values were adjusted to control the false discovery rate using the method of Benjamini and Yekutieli [85], by treating the combined set of *p*-values as a single family.

#### Training the final models

For building the models based upon the entire model development set that were used for the applicable descriptor importance analyses (see below), the same modelling protocol was repeated as described for each of the outer cross-validation training sets (see above), including selection of the best combination of hyperparameters via stratified cross-validation.

However, after checking that, as expected, the cross-validation results could be reproduced without scaling, the single descriptor Random Forest model that was selected for true external validation was rebuilt without scaling.

#### Descriptor importance analyses

Initially, descriptor importance values for Random Forest and logistic regression models were computed for models trained on the entire development set, after tuning the hyperparameters via cross-validation as per all models, in terms of the Gini importance measure [56] and coefficient magnitudes. Subsequently, for both methods, descriptor importance values were estimated using a permutation variable importance approach. Here, the importance of each descriptor was estimated as the decrease in balanced accuracy on a validation subset of the entire model development set, for a model trained on the remaining instances, with hyperparameter tuning and descriptor pre-processing in the usual fashion using the training set, when the validation set descriptor values were randomly shuffled [53–54]. Here, the validation subsets were the same five folds used to estimate model performance and the importance values were summarized across ten random seeds and all five folds.

As well as estimating the importance of the descriptors for both of these modelling algorithms, the importance of the descriptors was also estimated for a variation on the Random Forest algorithm, termed Cforest [57], in terms of its conditional variable importance measure [58]. A Cforest model was built on the entire model development set, following descriptor pre-processing in the usual fashion. The number of trees and number of descriptors randomly selected for evaluation at each split point were set to the options previously selected as part of hyperparameter tuning for the corresponding Random Forest model, with all other hyperparameters set to the recommended values for unbiased descriptor evaluation in Strobl et al. [57]. A Cforest model was built ten times, each time using a different random seed, and the variable importance values summarized over all ten models.

#### Applicability domain analysis for the final single descriptor model

For the final, externally validated single descriptor model, a simple applicability domain estimation was performed, whereby any instances for which this descriptor was less than the minimum or greater than the maximum values for the model development dataset were deemed to lie outside of the applicability domain. This simple range-based definition of the applicability domain is an established approach [66], but more complex methods have been proposed elsewhere in the QSAR literature [100].

#### Selection of data for external validation of the final single descriptor model

As discussed under “External validation results”, seven metal oxide ENMs were selected from the original set of data, used as the source of data for model development, extracted from the data records, exported from the Nanomaterial Biological-Interactions (NBI) Knowledgebase [26], and reported in the NanoHub online repository [81] as supplementary material for Karcher and co-workers [31]. These metal oxides were not included in the original model development dataset derived from these data as they were rejected according to the original selection criteria that needed to be applied for modelling the data using multiple descriptors. However, they were retained when these criteria were relaxed to (i) allow for the inclusion of minimally doped ENMs, (ii) allow for the selection of ENMs with missing numerical characterisation data used as input variables to the original, multi-descriptor models, and (iii) allow for the selection of materials tested at maximum dosage concentrations of less than 250 ppm. The latter point meant that those NMs not tested up to 250 ppm, for which a LOEL was not detected, may have been false negatives in terms of the categorical endpoint modelled herein, based upon whether the LOEL for excess mortality, in the period from 24 to 120 hpf, was less than or equal to 250 ppm.

In addition, the literature was searched for additional datasets to serve as approximate external validation sets. Only limited data were identified for metal oxide ENMs (including the metalloid oxide silicon dioxide) assessed for lethality against embryonic zebrafish and these were for endpoints that were not directly comparable to the categorical endpoint modelled herein. These limited additional datasets comprised the following: (1) LOEL data for three ENMs, assessed up to a concentration of 25 ppm, for cumulative lethality up to 120 hpf against embryonic zebrafish [101], where the detection of a LOEL value in this range of tested concentrations was deemed toxic and the failure to detect a LOEL “non-toxic”; (2) LC_50_ values (ppm), or imprecise estimates based upon a lower limit, for exposure up to 96 hpf, determined for 11 ENMs [102]; (3) LC_50_ values (micromolar) for two ENMs assessed at different timepoints [103], with the latter data points not being comparable to each other and hence unsuitable to assess the ability of the selected model to rank different metal oxide ENMs according to their lethality against embryonic zebrafish.

The first two of these additional datasets were evaluated to assess the ability of the prediction scores of the final Random Forest model, that is, the average proportion of toxic training set instances for the model development dataset assigned to the same leaf node as new instances, to be used for ranking the new ENMs with respect to the new endpoints. Good correlation was observed (Supporting Information File 1, Figure S16), in terms of the area under the receiver operator curve (AUC), between the model prediction scores and the categories assigned based upon the LOEL data reported for the three ENMs in the first of these datasets [101]. Moreover, no correlation was observed when simply ranking the ENMs according to the single selected descriptor, suggesting value was added by modelling the related endpoint (Supporting Information File 1, Figure S17). However, no correlation was obtained (Supporting Information File 1, Figure S18) between the model prediction scores and the (approximate) LC_50_ values retrieved for the second of these datasets [102], which could reflect greater differences to the modelled endpoint data, the lack of precision in some of these new datapoints, or the fact that some of the new ENMs were outside the domain.

## Supporting Information

File 1 PDF with two sections: (A) Additional results, (B) Detailed instructions for reproducing our results using code provided on Zenodo.

File 2 ZIP archive containing dataset files described in corresponding README files (not including a dataset file derived from the ChEMBL database).

File 3 ZIP archive containing a dataset file, described in the README file, derived from the ChEMBL database.
