# Supplementary material for: Identifying diverse metal oxide nanomaterials with lethal effects on embryonic zebrafish using machine learning
Source: Beilstein J Nanotechnol. 2021 Nov 29;12:1297–325. doi: 10.3762/bjnano.12.97 (PMC8649207; doi:10.3762/bjnano.12.97)
Supplement: File 1 — PDF with two sections: (A) Additional results, (B) Detailed instructions for reproducing our results using code provided on Zenodo. [file Beilstein_J_Nanotechnol-12-1297-s001.pdf]

BEILSTEIN JOURNAL OF NANOTECHNOLOGY

Supporting Information

for

Identifying diverse metal oxide nanomaterials with lethal effects on embryonic zebrafish using machine learning

Richard Liam Marchese Robinson, Haralambos Sarimveis, Philip Doganis, Xiaodong Jia, Marianna Kotzabasaki, Christiana Gousiadou, Stacey Lynn Harper and Terry Wilkins

Beilstein J. Nanotechnol. **2021,** *12,* 1297–1325. doi:10.3762/bjnano.12.97

PDF with two sections: (A) Additional results, (B) Detailed instructions for reproducing our results using code provided on Zenodo

License and Terms: This is a supporting information file under the terms of the Creative Commons Attribution License (https://creativecommons.org/licenses/by/4.0). Please note that the reuse, redistribution and reproduction in particular requires that the author(s) and source are credited and that individual graphics may be subject to special legal provisions.
The license is subject to the *Beilstein Journal of Nanotechnology* terms and conditions: (https://www.beilstein-journals.org/bjnano/terms)

Section A: Additional Results

Performance of All Models Obtained without Data Augmentation Using Consistent Cross-Validation Folds

Table S1: Average performance statistics obtained from cross-validation of Random Forest (RF) classification models and Logistic Regression (LR) models on consistent folds of the model development dataset. All models were built without data augmentation, using all calculated descriptors, unless noted otherwise. All results were obtained via tuning hyperparameters separately for each cross-validation training set, i.e., the multiple descriptor results are not expected to suffer from significant optimistic bias. However, the single descriptor (Pauling metal atom electronegativity) models were developed following descriptor importance analysis using the entire model development dataset. The performance in terms of each figure of merit is summarised as the arithmetic mean +/- standard error [median]. (The standard error of the mean is an underestimate of the uncertainty, as the cross-validation results are not entirely independent.) BA = balanced accuracy. MCC = Matthews Correlation Coefficient. AUC = area under the receiver-operator characteristic (ROC) curve.

Lethality Endpoint	Model	BA	MCC	AUC	Recall (Toxic)	Precision (Toxic)	Recall (Non-Toxic)	Precision (Non-Toxic)
24 hpf	RF	0.44 +/- 0.05 [0.50]	-0.12 +/- 0.10 [0.00]	0.36 +/- 0.10 [0.43]	0.13 +/- 0.13 [0.00]	0.07 +/- 0.07 [0.00]	0.75 +/- 0.12 [0.83]	0.67 +/- 0.05 [0.71]
120 hpf (excess lethality)	RF	0.71 +/- 0.08 [0.67]	0.45 +/- 0.17 [0.33]	0.76 +/- 0.09 [0.71]	0.53 +/- 0.12 [0.50]	0.67 +/- 0.14 [0.50]	0.88 +/- 0.05 [0.86]	0.83 +/- 0.05 [0.83]
	LR	0.74 +/- 0.11 [0.60]	0.48 +/- 0.21 [0.22]	0.70 +/- 0.14 [0.67]	0.63 +/- 0.15 [0.50]	0.63 +/- 0.15 [0.50]	0.85 +/- 0.07 [0.86]	0.85 +/- 0.06 [0.80]
	RF (single descriptor)	0.74 +/- 0.09 [0.75]	0.52 +/- 0.20 [0.65]	0.79 +/- 0.08 [0.83]	0.53 +/- 0.16 [0.50]	0.73 +/- 0.19 [1.00]	0.94 +/- 0.03 [1.00]	0.85 +/- 0.05 [0.86]
	LR (single descriptor)	0.73 +/- 0.10 [0.67]	0.43 +/- 0.19 [0.33]	0.73 +/- 0.11 [0.63]	0.63 +/- 0.15 [0.50]	0.57 +/- 0.12 [0.50]	0.82 +/- 0.05 [0.83]	0.85 +/- 0.06 [0.83]

Original 24 hpf lethality Results Obtained Using Stratified Cross-Validation Rather than Fixed Folds

Table S2: Average performance statistics obtained from stratified cross-validation of the original multiple descriptor Random Forest (RF) classification model of the 24 hpf lethality endpoint on the model development dataset. In contrast to the corresponding results reported in Table S1, the folds were those identified using stratification based upon the 24 hpf endpoint, rather than the same folds identified using stratification based upon the 120 hpf endpoint for consistent comparison of results. These results were obtained via nested cross-validation, i.e., are not expected to suffer from optimistic bias. The performance in terms of each figure of merit is summarised as the arithmetic mean +/- standard error [median]. (The standard error of the mean is an underestimate of the uncertainty, as the cross-validation results are not entirely independent.) BA = balanced accuracy. MCC = Matthews Correlation Coefficient. AUC = area under the receiver-operator characteristic (ROC) curve.

Lethality Endpoint	Model	BA	MCC	AUC	Recall (Toxic)	Precision (Toxic)	Recall (Non-Toxic)	Precision (Non-Toxic)
24 hpf	RF	0.52 +/- 0.07 [0.58]	0.09 +/- 0.15 [0.15]	0.49 +/- 0.14 [0.55]	0.30 +/- 0.08 [0.33]	0.43 +/- 0.16 [0.33]	0.74 +/- 0.08 [0.71]	0.72 +/- 0.03 [0.71]

Additional Data Augmentation Overall Performance Statistics for Modelling of the 120 hpf excess lethality endpoint

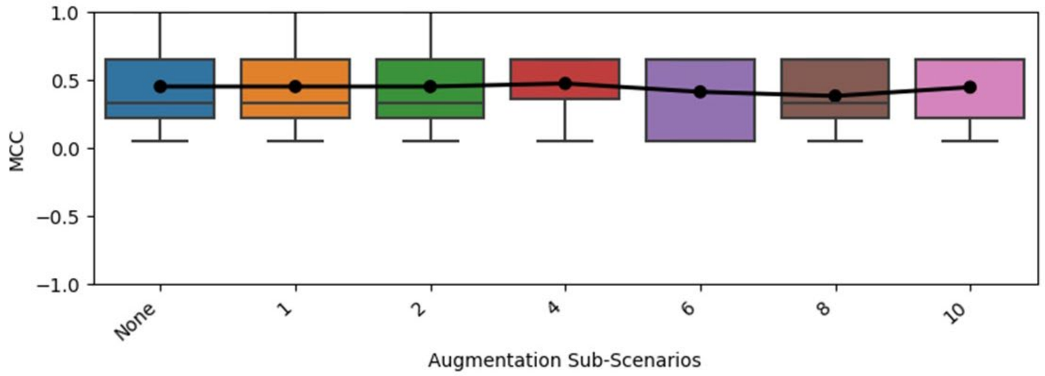

Figure S1: Cross-validated MCC obtained with Random Forest models for the 120 hpf excess lethality endpoint when the cross-validation training sets were supplemented with multiple noised replications (1 - 10 replications, with certain numbers skipped in keeping with literature precedence [1]) of themselves, compared to the results obtained with no data augmentation. The results across each test fold are summarized in terms of a boxplot, with the arithmetic mean result superimposed.

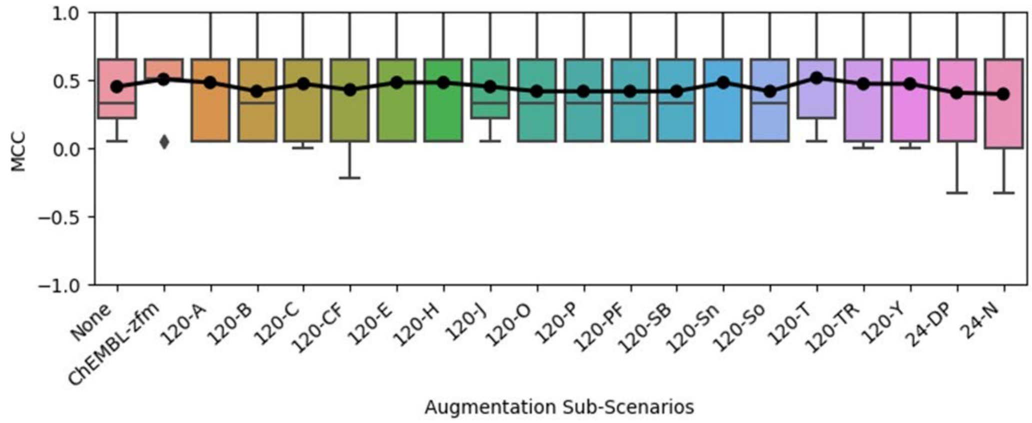

Figure S2: Cross-validated MCC obtained with Random Forest models for the 120 hpf excess lethality endpoint when the cross-validation training sets were supplemented with weighted alternative samples, compared to the results obtained with no data augmentation. Other than where molecular zebrafish lethality data from ChEMBL were treated as pseudo-coated ENM samples, all other alternative samples corresponded to the cross-validation training set with the modelled endpoint substituted with one of the sub-lethal endpoints. (See Endpoint Abbreviations under the Experimental section, in the main text, for an explanation of the 24 hpf and 120 hpf sub-lethal endpoint abbreviations.) The results across each test fold are summarized in terms of a boxplot, with the arithmetic mean result superimposed.

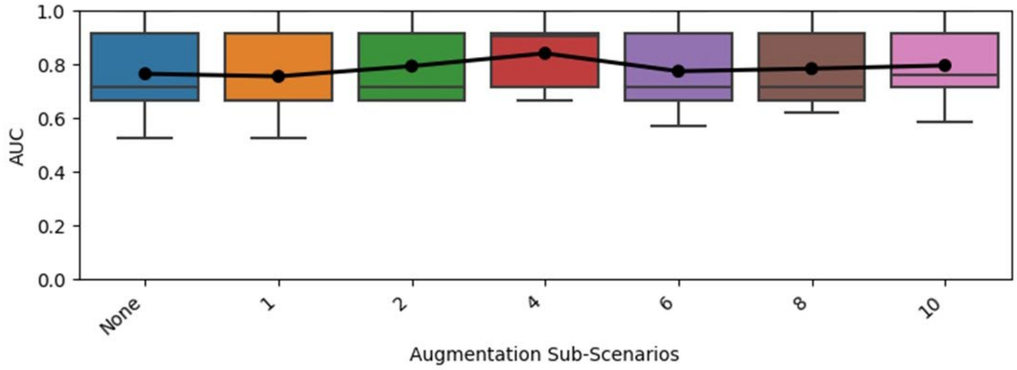

Figure S3: Cross-validated AUC obtained with Random Forest models for the 120 hpf excess lethality endpoint when the cross-validation training sets were supplemented with multiple noised replications (1 - 10 replications, with certain numbers skipped in keeping with literature precedence [1]) of themselves, compared to the results obtained with no data augmentation. The results across each test fold are summarized in terms of a boxplot, with the arithmetic mean result superimposed.

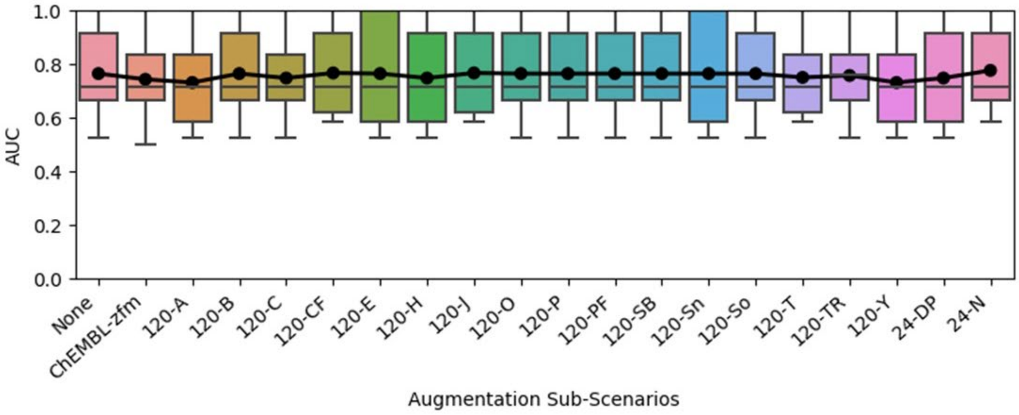

Figure S4: Cross-validated AUC obtained with Random Forest models for the 120 hpf excess lethality endpoint when the cross-validation training sets were supplemented with weighted alternative samples, compared to the results obtained with no data augmentation. Other than where molecular zebrafish lethality data from ChEMBL were treated as pseudo-coated ENM samples, all other alternative samples corresponded to the cross-validation training set with the modelled endpoint substituted with one of the sub-lethal endpoints. (See Endpoint Abbreviations under the Experimental section, in the main text, for an explanation of the 24 hpf and 120 hpf sub-lethal endpoint abbreviations.) The results across each test fold are summarized in terms of a boxplot, with the arithmetic mean result superimposed.

Data Augmentation Overall Performance Statistics for Modelling of the 24 hpf lethality endpoint

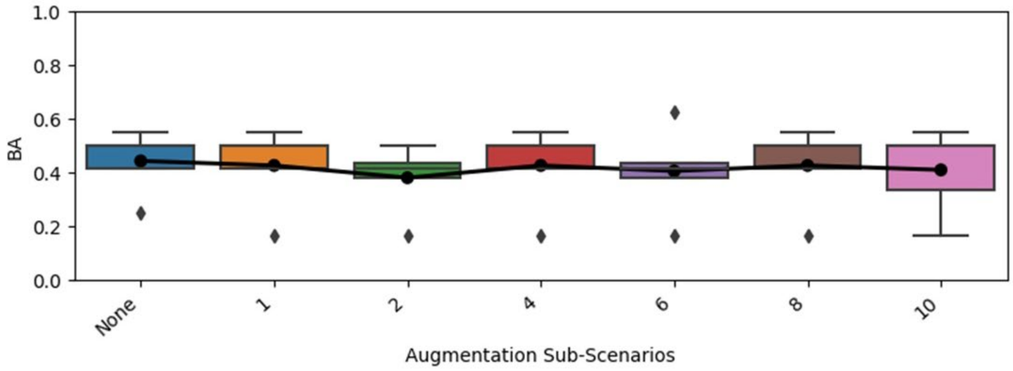

Figure S5: Cross-validated balanced accuracy (BA) obtained with Random Forest models for the 24 hpf lethality endpoint when the cross-validation training sets were supplemented with multiple noised replications (1 - 10 replications, with certain numbers skipped in keeping with literature precedence [1]) of themselves, compared to the results obtained with no data augmentation. The results across each test fold are summarized in terms of a boxplot, with the arithmetic mean result superimposed.

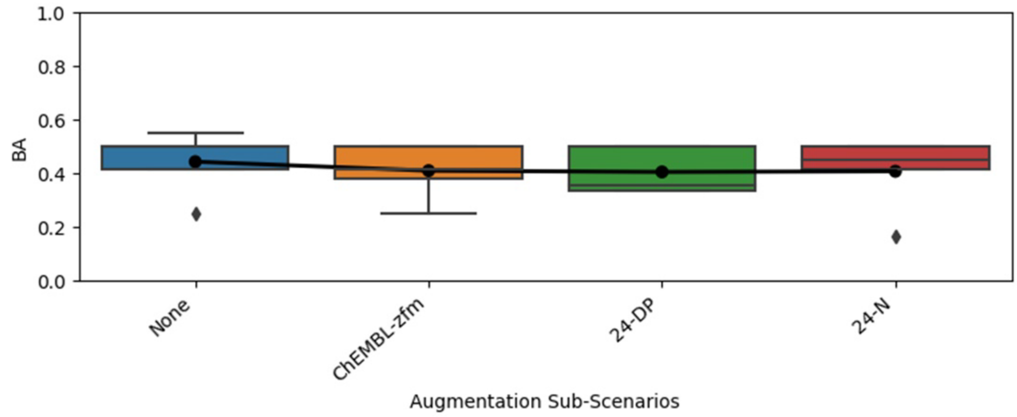

Figure S6: Cross-validated balanced accuracy (BA) obtained with Random Forest models for the 24 hpf lethality endpoint when the cross-validation training sets were supplemented with weighted alternative samples, compared to the results obtained with no data augmentation. Other than where molecular zebrafish lethality data from ChEMBL were treated as pseudo-coated ENM samples, all other alternative samples corresponded to the cross-validation training set with the modelled endpoint substituted with one of the sub-lethal endpoints. (See Endpoint Abbreviations under the Experimental section, in the main text, for an explanation of the 24 hpf and 120 hpf sub-lethal endpoint abbreviations.) The results across each test fold are summarized in terms of a boxplot, with the arithmetic mean result superimposed.

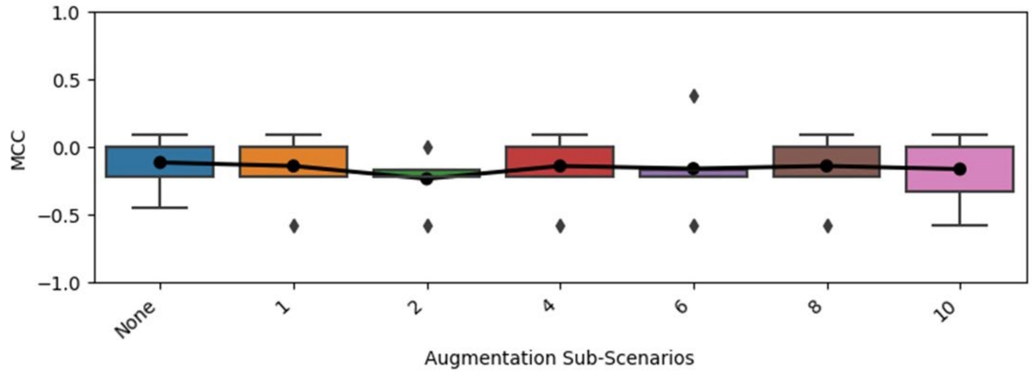

Figure S7: Cross-validated MCC obtained with Random Forest models for the 24 hpf lethality endpoint when the cross-validation training sets were supplemented with multiple noised replications (1 - 10 replications, with certain numbers skipped in keeping with literature precedence [1]) of themselves, compared to the results obtained with no data augmentation. The results across each test fold are summarized in terms of a boxplot, with the arithmetic mean result superimposed.

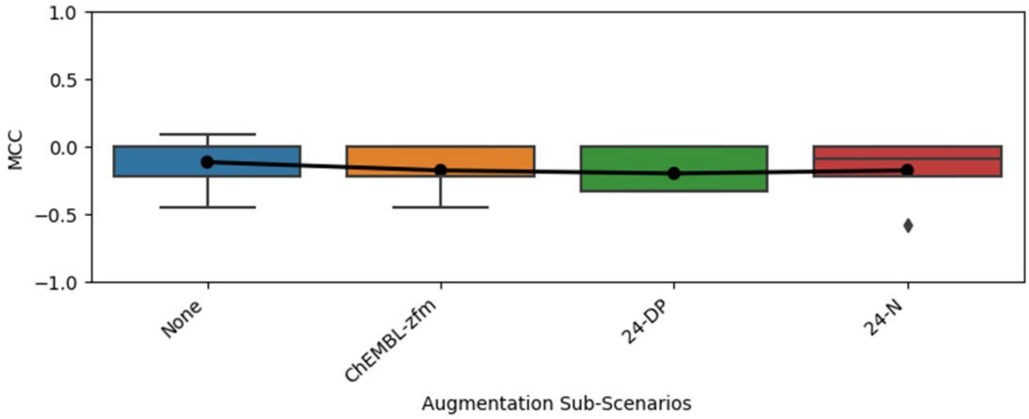

Figure S8: Cross-validated MCC obtained with Random Forest models for the 24 hpf lethality endpoint when the cross-validation training sets were supplemented with weighted alternative samples, compared to the results obtained with no data augmentation. Other than where molecular zebrafish lethality data from ChEMBL were treated as pseudo-coated ENM samples, all other alternative samples corresponded to the cross-validation training set with the modelled endpoint substituted with one of the sub-lethal endpoints. (See Endpoint Abbreviations under the Experimental section, in the main text, for an explanation of the 24 hpf and 120 hpf sub-lethal endpoint abbreviations.) The results across each test fold are summarized in terms of a boxplot, with the arithmetic mean result superimposed.

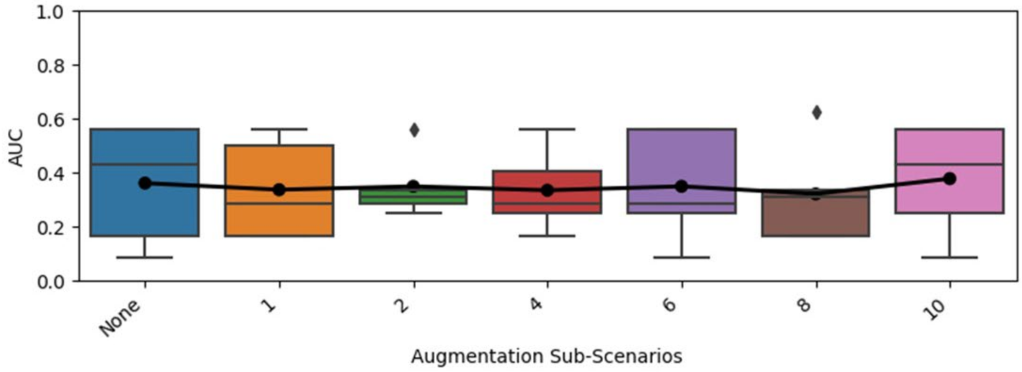

Figure S9: Cross-validated AUC obtained with Random Forest models for the 24 hpf lethality endpoint when the cross-validation training sets were supplemented with multiple noised replications (1 - 10 replications, with certain numbers skipped in keeping with literature precedence [1]) of themselves, compared to the results obtained with no data augmentation. The results across each test fold are summarized in terms of a boxplot, with the arithmetic mean result superimposed.

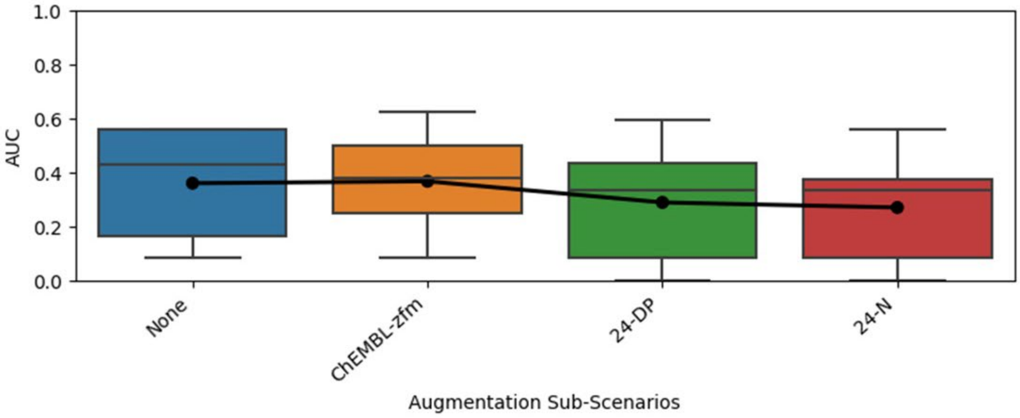

Figure S10: Cross-validated AUC obtained with Random Forest models for the 24 hpf lethality endpoint when the cross-validation training sets were supplemented with weighted alternative samples, compared to the results obtained with no data augmentation. Other than where molecular zebrafish lethality data from ChEMBL were treated as pseudo-coated ENM samples, all other alternative samples corresponded to the cross-validation training set with the modelled endpoint substituted with one of the sub-lethal endpoints. (See Endpoint Abbreviations under the Experimental section, in the main text, for an explanation of the 24 hpf and 120 hpf sub-lethal endpoint abbreviations.) The results across each test fold are summarized in terms of a boxplot, with the arithmetic mean result superimposed.

Additional Changes in Overall Performance Statistics Due to Weighted Alternative Samples Data Augmentation Plotted Against the Weighting Applied for Modelling of the 120 hpf excess lethality endpoint

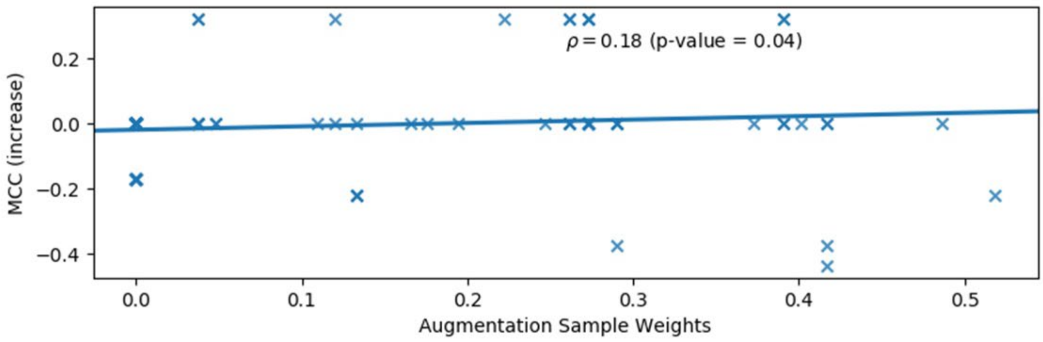

Figure S11: The change in cross-validated MCC obtained with Random Forest models for the 120 hpf excess lethality endpoint when the cross-validation training sets were supplemented with alternative samples, derived via replacing the training set sample endpoint values with the endpoint values for sub-lethal endpoints measured for the same materials, plotted against the weight given to these alternative samples. Here, the weight assigned was either the MCC correlation measure, if this was positive, or zero. The Spearman rank correlation coefficient is shown, along with the corresponding one-tail p-value for the null hypothesis that this is zero, i.e., that there is no correlation between the change in performance and the relationship between the modelled and sub-lethal endpoint data used for data augmentation, with the alternative hypothesis being a positive rank correlation.

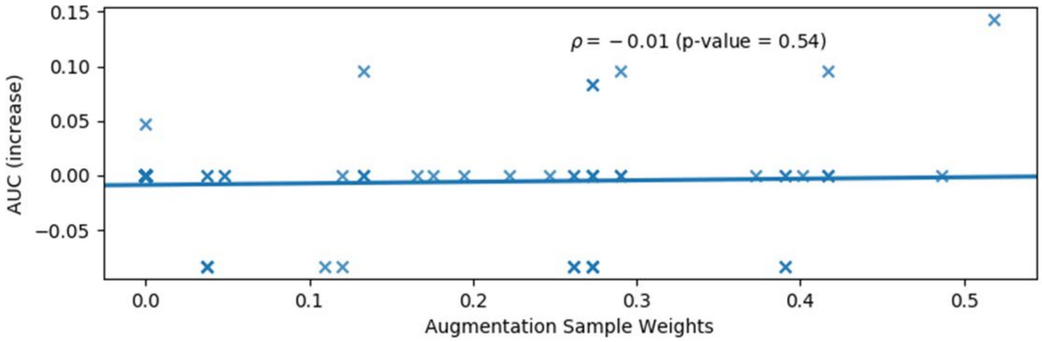

Figure S12. The change in cross-validated AUC obtained with Random Forest models for the 120 hpf excess lethality endpoint when the cross-validation training sets were supplemented with alternative samples, derived via replacing the training set sample endpoint values with the endpoint values for sub-lethal endpoints measured for the same materials, plotted against the weight given to these alternative samples. Here, the weight assigned was either the MCC correlation measure, if this was positive, or zero. The Spearman rank correlation coefficient is shown, along with the corresponding one-tail p-value for the null hypothesis that this is zero, i.e., that there is no correlation between the change in performance and the relationship between the modelled and sub-lethal endpoint data used for data augmentation, with the alternative hypothesis being a positive rank correlation.

Changes in Overall Performance Statistics Due to Weighted Alternative Samples Data Augmentation Plotted Against the Weighting Applied for Modelling of the 24 hpf lethality endpoint

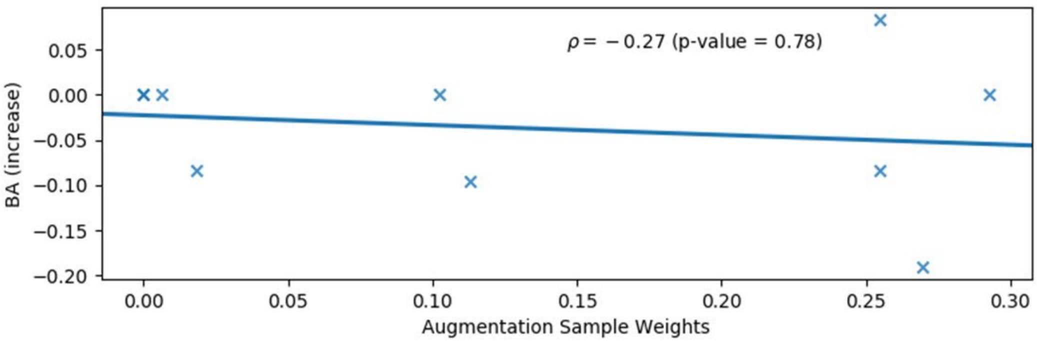

Figure S13: The change in cross-validated balanced accuracy (BA) obtained with Random Forest models for the 24 hpf lethality endpoint when the cross-validation training sets were supplemented with alternative samples, derived via replacing the training set sample endpoint values with the endpoint values for sub-lethal endpoints measured for the same materials, plotted against the weight given to these alternative samples. Here, the weight assigned was either the MCC correlation measure, if this was positive, or zero. The Spearman rank correlation coefficient is shown, along with the corresponding one-tail p-value for the null hypothesis that this is zero, i.e., that there is no correlation between the change in performance and the relationship between the modelled and sub-lethal endpoint data used for data augmentation, with the alternative hypothesis being a positive rank correlation.

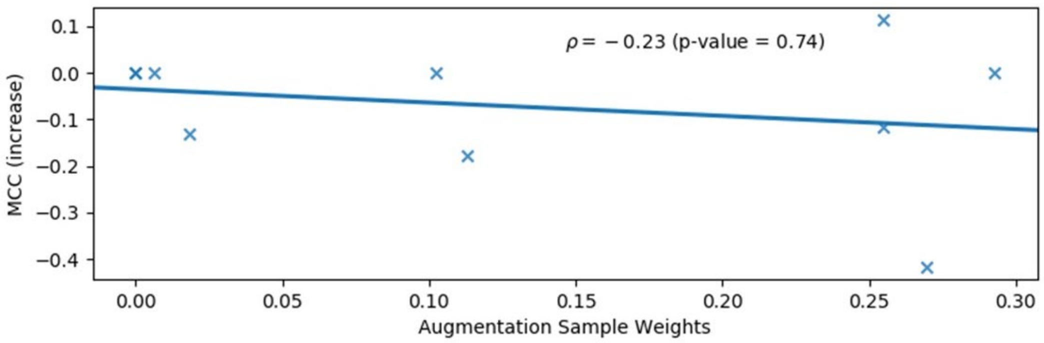

Figure S14: The change in cross-validated MCC obtained with Random Forest models for the 24 hpf lethality endpoint when the cross-validation training sets were supplemented with alternative samples, derived via replacing the training set sample endpoint values with the endpoint values for sub-lethal endpoints measured for the same materials, plotted against the weight given to these alternative samples. Here, the weight assigned was either the MCC correlation measure, if this was positive, or zero. The Spearman rank correlation coefficient is shown, along with the corresponding one-tail p-value for the null hypothesis that this is zero, i.e., that there is no correlation between the change in performance and the relationship between the modelled and sub-lethal endpoint data used for data augmentation, with the alternative hypothesis being a positive rank correlation.

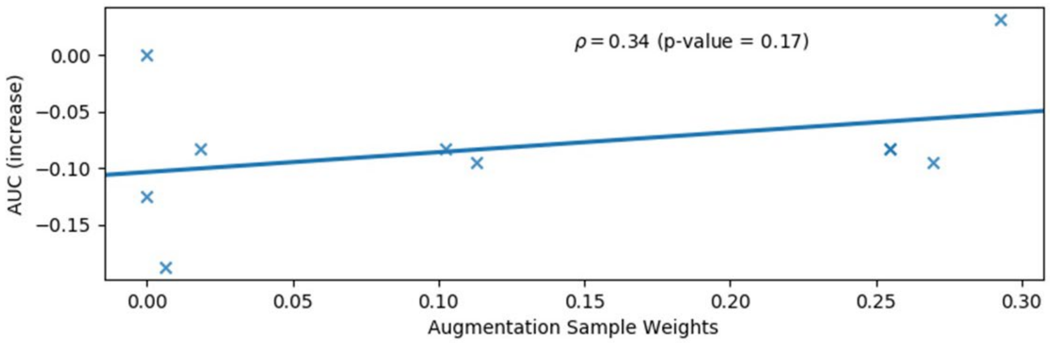

Figure S15: The change in cross-validated AUC obtained with Random Forest models for the 24 hpf lethality endpoint when the cross-validation training sets were supplemented with alternative samples, derived via replacing the training set sample endpoint values with the endpoint values for sub-lethal endpoints measured for the same materials, plotted against the weight given to these alternative samples. Here, the weight assigned was either the MCC correlation measure, if this was positive, or zero. The Spearman rank correlation coefficient is shown, along with the corresponding one-tail p-value for the null hypothesis that this is zero, i.e., that there is no correlation between the change in performance and the relationship between the modelled and sub-lethal endpoint data used for data augmentation, with the alternative hypothesis being a positive rank correlation.

Additional External Validation Results for the Final Single Descriptor Model Compared to Directly Ranking Using the Single Descriptor

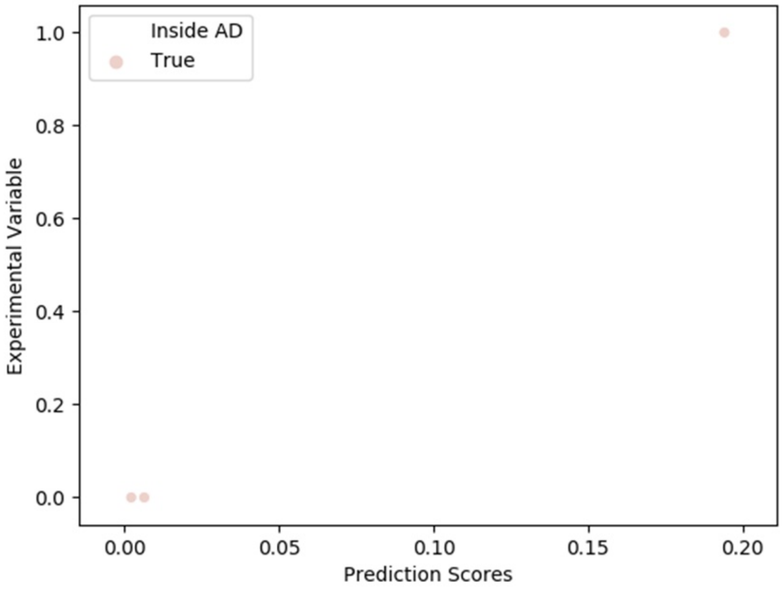

Figure S16: Correlation (AUC = 1) between the experimental variable (toxic = 1, non-toxic = 0, based upon cumulative lethality up to 120 hpf against embryonic zebrafish - where the detection of a LOEL value in this range of tested concentrations was deemed toxic and the failure to detect a LOEL "non-toxic") and the prediction score (average percentage of toxic training set ENMs assigned to the same leaf node as the predicted ENM across all trees) for the selected Random Forest model, trained on the entire model development dataset, using only the Pauling metal atom electronegativity descriptor. These approximate external test data were retrieved from George et al. [2].

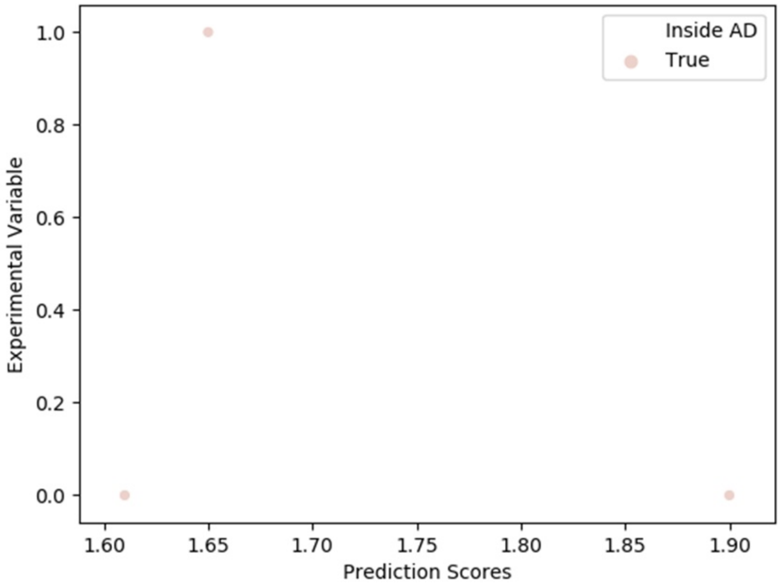

Figure S17: Correlation (AUC = 0.5) between the experimental variable (toxic = 1, non-toxic = 0, based upon cumulative lethality up to 120 hpf against embryonic zebrafish - where the detection of a LOEL value in this range of tested concentrations was deemed toxic and the failure to detect a LOEL "non-toxic") and Pauling metal atom electronegativity descriptor (the "prediction score"). These approximate external test data were retrieved from George et al. [2].

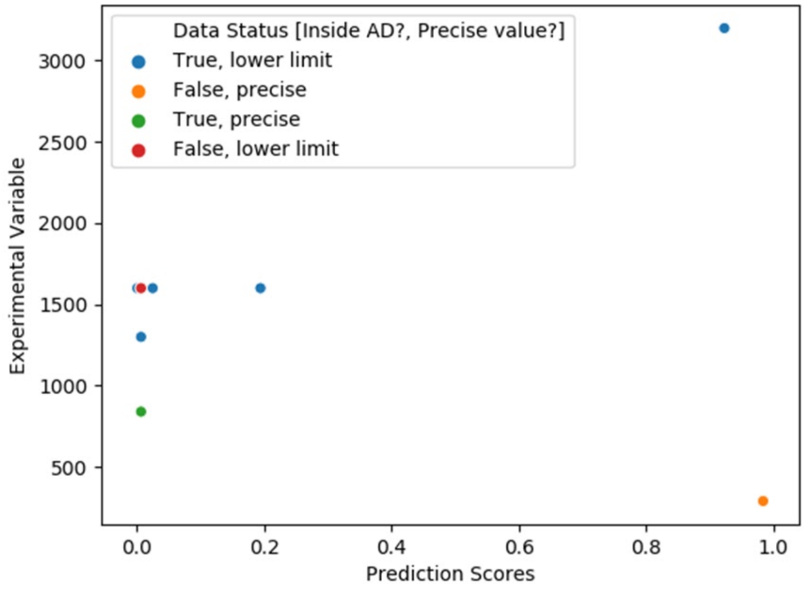

Figure S18: Correlation between the experimental variable (LC_{50} values (ppm), or imprecise estimates based upon a lower limit, for exposure up to 96 hpf) and the prediction score (average percentage of toxic training set ENMs assigned to the same leaf node as the predicted ENM across all trees) for the selected Random Forest model, trained on the entire model development dataset, using only the Pauling metal atom electronegativity descriptor. These approximate external test data were retrieved from Kovriznych et al. [3].

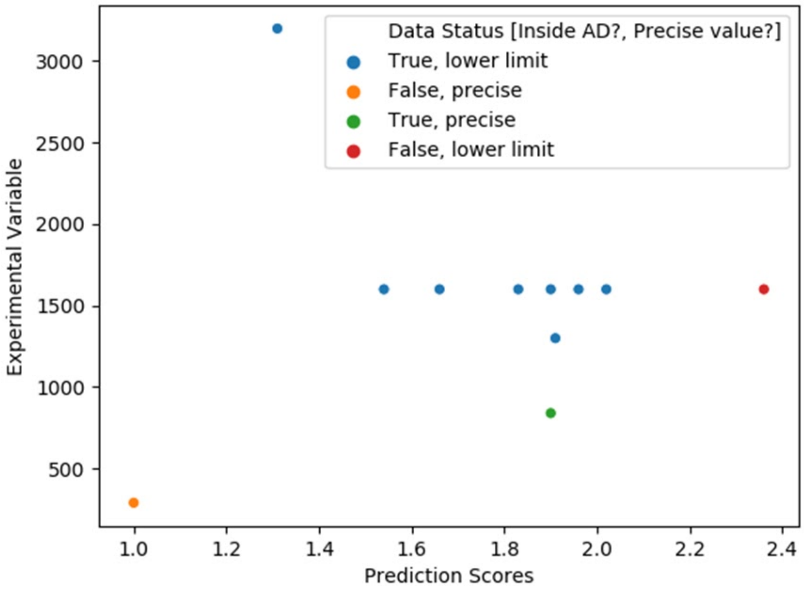

Figure S19: Correlation between the experimental variable (LC_{50} values (ppm), or imprecise estimates based upon a lower limit, for exposure up to 96 hpf) and Pauling metal atom electronegativity descriptor (the "prediction score"). These approximate external test data were retrieved from Kovriznych et al. [3].

Section B: How to Reproduce Our Results

This guide assumes the calculations are being performed on a machine running Windows.

1. Download the code archive from Zenodo [4]
    a. Extract the folder 'CodeArchive' -> C:\CodeArchive
2. Install Anaconda3-2019.07-Windows-x86_64.exe from https://repo.anaconda.com/archive/ (last accessed 06/03/2021)
    a. Run this as an administrator
    b. Close other applications when prompted
    c. Select "Add Ananconda to the system PATH" when prompted
    d. Try typing 'python' in a new command prompt to check the installation
3. Create the default Python environment: biorima_3
    a. Navigate to C:\CodeArchive\EnvironmentFiles in the command prompt
    b. conda env create -f 14.10.2019_biorima_3_conda_env_export.yml
    c. Check this environment has been created via activating it: conda activate biorima_3
    d. Quit this environment: conda deactivate
4. Create the Python environment used for the permutation variable importance calculations: permutation_importance_biorima_3
    a. Navigate to C:\CodeArchive\EnvironmentFiles\perm_imp_specific_env_file in the command prompt
    b. conda env create -f permutation_importance_biorima_3.yml
    c. Check this new environment has been created via activating it: conda activate permutation_importance_biorima_3
    d. Quit this environment: conda deactivate
5. Install R-3.6.3-win.exe from https://cloud.r-project.org/bin/windows/base/old/3.6.3/ (last accessed 06/03/2021)
    a. Run this as administrator
    b. Close other applications when prompted
6. Install the party package, including the Cforest implementation, and its dependencies
    a. These were installed via entering this command in the R GUI: install.packages(c("party"))
    b. However, to install the exact same versions, these may need to be installed from ZIP files, which could be located here: https://cran.microsoft.com/snapshot/2020-05-24/web/packages/ (last accessed 06/03/2021)
    c. The versions of these packages installed for the current work were as follows: party_1.3-4.zip, TH.data_1.0-10.zip, libcoin_1.0-5.zip, matrixStats_0.56.0.zip, multcomp_1.4-13.zip, mvtnorm_1.1-0.zip, modeltools_0.2-23.zip, strucchange_1.5-2.zip, coin_1.3-1.zip, zoo_1.8-8.zip, sandwich_2.5-1.zip
7. Install MOPAC2016_with_a_window_for_WINDOWS_64_bit.zip from http://openmopac.net/Download_MOPAC_Executable_Step2.html (last accessed 06/03/21)
    a. Extract the ZIP and follow the installation instructions, including guidance on obtaining a license key, from "Installation instructions.txt"
8. Download the NBI Knowledgebase data files used to derive the model development dataset (using an automated workflow), as well as one external test set (filtering operations were applied manually following the derivation of LOEL values via the automated workflow), from the Supporting Information of Karcher et al. [5] on NanoHUB [6]
    a. Extract all nbi_[ID].xls files into the following folder: C:\CodeArchive\BioRima_calc_UoL.final\BioRima_calc\NBI_data\KarcherEtAl_2016_nanoHUB\NBI_SourceData_12082015

9. Start a new Windows command prompt
10. conda activate biorima_3
11. Navigate to C:\CodeArchive\BioRima_calc_UoL.final\BioRima_calc\BioRima_rlmr\prototype_da_models_workflow
12. python run_nbik_da_prototype_models_workflow.py
    a. This will generate the model development dataset, calculate all descriptors and perform all multiple descriptor Random Forest modelling calculations, including with data augmentation.
    b. This will also generate cross-validation statistics for modelling the 24 hpf lethality endpoint, using both independently generated folds from stratified cross-validation and the fixed folds corresponding to the stratified cross-validation folds based upon the 120 hpf excess lethality endpoint.
    c. This will additionally generate cross-validation statistics for modelling the 120 hpf excess lethality endpoint, with and without data augmentation.
    d. If problems are encountered when trying to compute Absolv descriptors:
        i. Navigate to C:\CodeArchive\BioRima_calc_UoL.final\BioRima_calc\BioRima_rlmr\Calc_approx_Abrahams_descs and run this command: python build_eval_apply_approxAbraham_descs.py
    e. Due to recent ChEMBL [7,8] web-service [9] updates, the version of chembl-webresource-client installed as part of the biorima_3 environment no longer works and this script will fail if this issue is not fixed
        i. As a workaround, the file containing the ChEMBL data retrieved when this script was originally run can be extracted from the Supporting Information ZIP archive: ChEMBL_zfm_precursor.csv
        ii. This file should be placed inside this folder: C:\CodeArchive\BioRima_calc_UoL.final\BioRima_calc\prototype_da_model_files\data_aug_input
        iii. To enable the script to run, comment out the following lines:
            1. Line 32: from chembl_webresource_client.new_client import new_client [-> #from chembl_webresource_client.new_client import new_client]
            2. Line 1350: downloadRelevantChEMBLdata(data_aug_alt_samples_precursor_file) [-> #downloadRelevantChEMBLdata(data_aug_alt_samples_precursor_file)]
13. python analyse_nbik_da_prototype_model_results.py
    a. This will perform additional analysis of the multiple descriptor Random Forest modelling results on the model development dataset.
14. Navigate to C:\CodeArchive\BioRima_MarcheseRobinsonEtAlExtraCalc\logreg_rev_calc
15. python LogisticReg_Nested_CV_BioRima_MarcheseRobinsonEtAl.py
    a. This will generate the cross-validated, multiple descriptor Logistic Regression results.
16. Navigate to C:\CodeArchive\BioRima_MarcheseRobinsonEtAlExtraCalc
17. python compare_RF_and_LR_results.py
    a. This will re-generate cross-validation performance statistics on the model development dataset for the multiple descriptor Random Forest model of the 120 hpf excess lethality endpoint.
    b. It will generate the corresponding statistics for the multiple descriptor Logistic Regression model.
18. Navigate to C:\CodeArchive\BioRima_MarcheseRobinsonEtAlExtraCalc\cforest_var_imp

19. python run_multiple_cforest_calculations_and_analysis.py
    a. This will perform the Cforest descriptor importance analyses.
20. Navigate to C:\CodeArchive\BioRima_MarcheseRobinsonEtAlExtraCalc\rf_logrev_var_imp_further_analysis
21. python run_rf_logrev_original_imp_analysis.py
    a. This will perform the default Random Forest (Gini) and Logistic Regression (coefficient magnitude) descriptor importance analyses.
22. conda deactivate
23. conda activate permutation_importance_biorima_3
24. python run_rf_logrev_perm_imp_analysis.py
    a. This will perform the permutation variable importance analyses.
25. conda deactivate
26. conda activate biorima_3
27. Navigate to C:\CodeArchive\BioRima_MarcheseRobinsonEtAlExtraCalc\logreg_rev_calc\single_var
28. python single_var_LogisticReg_RF_Nested_CV_BioRima_MarcheseRobinsonEtAl.py
    a. This generates the cross-validated results for the single descriptor (Pauling metal atom electronegativity, identified via the previously described descriptor importance analyses) Logistic Regression and Random Forest models.
29. python single_var_compare_RF_and_LR_results.py
    a. This computes the cross-validated performance statistics for the single descriptor Logistic Regression and Random Forest models.
30. Navigate to C:\CodeArchive\BioRima_MarcheseRobinsonEtAlExtraCalc\logreg_rev_calc\single_var\check_no_scale_rf
31. python check_rf_single_var_model_CV_preds_unaffected_by_scaling.py
    a. This will re-generates the cross-validated results and re-builds the single descriptor Random Forest model without scaling and mean centering the descriptor based upon the training set, which allows for facile application to an external test set, i.e., no descriptor scaling is required.
    b. This also checks that the modelling results are, as expected for Random Forest, unaffected by scaling and mean centering.
        i. In practice, some minor changes in the scores assigned for cross-validated predictions were observed. This is expected to be an artefact of numerical approximations.
        ii. However, the cross-validated performance statistics - obtained using the next script - were unchanged.
32. python single_var_eval_RF_unscaled_results.py
    a. This computes the cross-validated performance statistics for the single descriptor Random Forest model without scaling and mean centering.
33. Navigate to C:\CodeArchive\BioRima_MarcheseRobinsonEtAlExtraCalc\ext_val_inc_AD_definition
34. Copy the folders with the external test datasets from the Supporting Information ZIP archive into \prep_single_var_ext_test\
35. python compute_ext_perf_stats.py

References

(1) Cortes-Ciriano, I.; Bender, A. *J. Chem. Inf. Model.* **2015**, *55* (12), 2682–2692. doi:10.1021/acs.jcim.5b00570

(2) George, S.; Xia, T.; Rallo, R.; Zhao, Y.; Ji, Z.; Lin, S.; Wang, X.; Zhang, H.; France, B.; Schoenfeld, D.; Damoiseaux, R.; Liu, R.; Lin, S.; Bradley, K. A.; Cohen, Y.; Nel, A. E. *ACS Nano* **2011**, *5* (3), 1805–1817. doi:10.1021/nn102734s

(3) Kovrižnych, J. *Interdiscip. Toxicol.* **2013**, *6* (2), 67–73. doi:10.2478/intox-2013-0012

(4) Marchese Robinson, R. L.; Sarimveis, H. Code for “Identifying diverse metal oxide nanomaterials with lethal effects on embryonic zebrafish using Machine Learning” https://zenodo.org/record/4681184 (accessed Jun 5, 2021). doi:10.5281/zenodo.4681184

(5) Karcher, S. C.; Harper, B. J.; Harper, S. L.; Hendren, C. O.; Wiesner, M. R.; Lowry, G. V. *Environ. Sci. Nano* **2016**, *3* (6), 1280–1292. doi:10.1039/C6EN00273K

(6) Karcher, S. Informatics Tool to Explore the Nanomaterial-Biological Interactions Knowledgebase https://nanohub.org/resources/23991/supportingdocs (accessed Aug 1, 2019)

(7) EMBL-EBI. ChEMBL Database https://www.ebi.ac.uk/chembl (accessed Nov 20, 2019)

(8) Gaulton, A.; Hersey, A.; Nowotka, M.; Bento, A. P.; Chambers, J.; Mendez, D.; Mutowo, P.; Atkinson, F.; Bellis, L. J.; Cibrián-Uhalte, E.; Davies, M.; Dedman, N.; Karlsson, A.; Magariños, M. P.; Overington, J. P.; Papadatos, G.; Smit, I.; Leach, A. R. *Nucleic Acids Res.* **2017**, *45* (D1), D945–D954. doi:10.1093/nar/gkw1074

(9) Davies, M.; Nowotka, M.; Papadatos, G.; Dedman, N.; Gaulton, A.; Atkinson, F.; Bellis, L.; Overington, J. P. *Nucleic Acids Res.* **2015**, *43* (W1), W612–W620. doi:10.1093/nar/gkv352
